# High CBD extract (CBD-X) modulates inflammation and immune cell activity in rheumatoid arthritis

**DOI:** 10.3389/fimmu.2025.1599109

**Published:** 2025-07-10

**Authors:** Miran Aswad, Antonina Pechkovsky, Narmeen Ghanayiem, Haya Hamza, Igal Louria-Hayon

**Affiliations:** ^1^ The Shanti Center For Medical Cannabis Research, Rambam Health Care Campus, Haifa, Israel; ^2^ Clinical Research Institute at Rambam (CRIR), Rambam Health Care Campus, Haifa, Israel

**Keywords:** CBD-X, rheumatoid arthritis, inflammation, neutrophils, CIA, CAIA

## Abstract

**Introduction:**

Rheumatoid arthritis (RA) is a debilitating autoimmune disease affecting approximately 1% of the global population and is associated with significant morbidity and mortality. Given the known anti-inflammatory effects of cannabinoids, we investigated the therapeutic potential of a high-CBD extract, termed CBD-X, by assessing its effects on immune cells and disease progression. This study investigates the therapeutic potential of a high-CBD extract (CBD-X) in RA.

**Methods:**

We evaluated the effects of CBD-X on cells involved in RA pathogenesis using macrophages and primary human neutrophils as ex vivo models. In addition, two murine models of RA were applied: collagen-induced arthritis (CIA) and collagen antibody-induced arthritis (CAIA).

**Results:**

Ex vivo experiments demonstrated that CBD-X inhibited the secretion of pro-inflammatory cytokines, including IL-1β from macrophages and IL-8, IL-6, and TNF-α from human neutrophils, suggesting its potential to modulate inflammatory responses. Moreover, CBD-X attenuated NF-κB p65 and Akt phosphorylation downstream LPS-activation signal in neutrophils. To further evaluate its therapeutic effects, we employed two murine models of RA: collagen-induced arthritis (CIA) and collagen antibody-induced arthritis (CAIA). In both models, CBD-X treatment resulted in a significant reduction of leukocyte levels in the blood, primarily through the suppression of neutrophil and monocyte populations, which play a central role in RA pathogenesis. Additionally, CBD-X reduced neutrophil migration to the joints in the CAIA model, highlighting its potential to alleviate joint inflammation. Furthermore, it modulated the neutrophil-to-macrophage ratio (NMR), an important marker of RA progression, an effect that was not observed with dexamethasone treatment, suggesting a distinct mechanism of immune regulation. Notably, CBD-X promoted the pro-resolving macrophages to the rheumatic joints. Importantly, CBD-X exerted its anti-inflammatory effect by downregulating TNF-α and MCP-1 while upregulating IL-10, a key anti-inflammatory cytokine involved in immune homeostasis.

**Discussion:**

These findings indicate that CBD-X has a significant potential as a therapeutic agent for RA, offering a promising approach to modulate immune responses and reduce inflammation in RA patients.

## Introduction

Rheumatoid arthritis (RA) is a debilitating autoimmune and inflammatory disease that affects approximately 1% of the global population and is associated with significant morbidity and mortality (1). A hallmark of RA is persistent synovitis, which results from the continuous influx of immune cells into the joints. In this process, effector T cells, along with B cells and other innate immune cells, form a complex network that promotes the production of pro-inflammatory cytokines. These cytokines activate resident fibroblast-like synoviocytes, contributing to cartilage and bone damage (2).

Innate immune cells, including neutrophils, mast cells, and macrophages, play a pivotal role in the development of synovitis. Macrophages, in particular, are central to RA pathophysiology, serving as the main source of pro-inflammatory cytokines (such as TNF-α, IL-1, and IL-6) and small-molecule mediators of inflammation, including reactive oxygen species, nitrogen intermediates, and prostanoids. These molecules perpetuate chronic inflammation, driving the tissue destruction and pain characteristic of RA (3).

Of all cells implicated in the pathology of rheumatoid arthritis (RA), neutrophils possess the greatest cytotoxic potential, owing to their ability to release degradative enzymes and reactive oxygen species. Neutrophils also contribute to the cytokine and chemokine cascades that accompany inflammation, and regulate immune responses via cell–cell interactions (4).

Early diagnosis and intervention in RA are crucial, as they can prevent or slow down the progression of joint damage in up to 90% of patients, thus preventing irreversible disability. Outcomes have improved with the advent of early therapy using disease-modifying antirheumatic drugs (DMARDs), with methotrexate being the first-line treatment. If this fails, biologic agents, including tumor necrosis factor (TNF) inhibitors, or Janus kinase inhibitors, often in combination with methotrexate, are employed to control disease activity (5).

More recently, there has been growing interest in the potential therapeutic role of cannabis, particularly cannabidiol (CBD), in managing inflammation. CBD is a potent immune-modulator with immune-suppressive properties in sterile inflammation and immune-protective effects in viral infections (6).

Cannabinoids, including CBD, have demonstrated anti-inflammatory effects in conditions like severe COVID-19 and may help prevent disease progression from mild to severe stages (7). Furthermore, CBD has been recognized for its beneficial properties as a potential treatment for rheumatoid diseases, owing to its anti-inflammatory and analgesic effects (8).

CBD, a non-psychoactive cannabis component, has been shown to be an effective oral anti-arthritic therapeutic in murine collagen-induced arthritis (9). In an osteoarthritis model, CBD effectively alleviated pain and claudication, significantly reducing inflammatory cytokine levels (IFN-γ, IL-1β, TNF-α) in serum and synovial fluid, as well as ameliorating arthritic pathological changes (10).

In addition to these findings, a high-CBD extract (CBD-X) has been identified as an effective agent in downregulating cytokine storms both systemically and locally in inflamed lungs (11). Furthermore, a recent study highlighted the significant potential of CBD-X in treating asthma (12).

In this study, we investigated the effects of a high-CBD extract (CBD-X) on macrophages and neutrophils ex vivo, both of which are critical contributors to rheumatoid arthritis (RA) pathogenesis. To further evaluate its therapeutic potential, we employed two murine models of RA. First, we used a collagen-induced arthritis (CIA) model to assess the impact of CBD-X on leukocyte, neutrophil and monocyte counts, as well as inflammatory cytokine levels, including MCP-1, in the blood. Second, we utilized an alternative model of RA induced by ArthritoMab, a cocktail of monoclonal antibodies targeting collagen II (CII), to investigate the effects of CBD-X on cell counts and cytokine levels in both blood and joint tissues. Our findings aim to provide preclinical evidence supporting the anti-inflammatory properties of CBD-X and its potential clinical application as a therapeutic agent for RA patients.

## Materials and methods

### Reagents

ELISA kits for mouse IL- 1β, TNF- α, MCP-1, IL- 10 and human IL-8, IL-6 and TNF- α were purchased from Biolegend (CA, USA).

RBC Lysis Buffer (10X), TruStain FcX™ (rat anti-mouse CD16/32, IgG2a, λ) and APC anti-mouse CD45 (rat, IgG2b, κ, clone 30/F11), Brilliant Violet 421 anti-mouse CD11b (Rat IgG2b, κ), PE anti-mouse F4/80 (Rat IgG2a, κ), Brilliant Violet 785 anti-mouse Ly-6G (Rat IgG2a, κ, clone 1A8) and Brilliant Violet 421 anti-mouse Ly-6C (Rat IgG2c, κ) were obtained from BioLegend (CA, USA).

ArthritoMab was purchased from MD Bioproducts (Zurich, Switzerland).

Dexamethasone was purchased from R&D systems (MN, USA).

The medium Dulbecco’s Modified Eagle Medium (DMEM) for RAW 264.7 cells was obtained from Sartorius (Beit Haemek, Israel) and the medium X-VIVO 15 with gentamicin and phenol red for neutrophil cells was obtained from Lonza (Basel, Switzerland).

Human Neutrophil Isolation kit was obtained from STEMCELL Technologies (Vancouver, Canada).

Lipopolysaccharide (LPS) was obtained from Santa Cruz (CA, USA).

Fetal bovine serum (FBS), glutamine and penicillin and 100 U/ml streptomycin were obtained from Biological Industries (Beit Haemek, Israel).

Acetic acid, Chicken Collagen Type II, Freund′s Adjuvant, Complete and Freund′s Incomplete Adjuvant from Merck (Darmstadt, Germany). Chicken collagen with CFA was prepared as follows: chicken collagen was dissolved in 0.01N acetic acid for a final concentration of 4 mg/ml. Then 0.5 ml of collagen was added to 3.5 ml of CFA (1 ml CFA contains 1 mg Mycobacterium butyricum). Additionally, 1 ml of chicken collagen was added to 3 ml Incomplete Freund Adjuvant (IFA) as a booster.

### Cannabis extracts

Cannabis extracts were kindly provided by Raphael Pharmaceutical, Inc (Nevada, USA). The strains were cultivated and grown by WOLC- Way Of Life Cannabis (Israel). An analytical characterization of CBD-X extract for three batches (20-851, 20–852 and 20-853) tested by Nextar chempharma solutions are shown (**Supplementary Figure S16**). The test show the percentage of the active cannabinoids CBG, CBD, CBN and THC in each batch. In average, the purity numbers are 0.32% CBG, 35.8% CBD, ND CBN and 1.7% THC.

### Cell culture

The RAW 264.7 mouse macrophages were cultured in DMEM supplemented with 10% heat-inactivated FBS, 3 mM glutamine and 100 U/ml penicillin and 100 U/ml streptomycin at 37°C under a humidified atmosphere of 5% CO2. Subcultures are prepared by scraping cells from floor of dishes every two days and diluting to 1 x 10^6 cells/20 ml. Medium renewal is 2 to 3 times per week. In all experiments, cells were allowed to acclimate overnight before treatments.

### Human peripheral blood samples

Human peripheral blood samples were obtained from the Israeli National Biobank for Research (MIDGAM) at Rambam Health Care Campus. The experiments were authorized by the Helsinki Committee at Rambam health Care Campus (Authorization No. 0442–20 RMB).

### Mice

C57BL/6 mice were obtained at the age of 8 to 10 weeks from Envigo, Israel. All mice were housed at a barrier/free and specific pathogen/free animal facility at the Pre-Clinical Research Authority, Technion-Israel Institute of Technology in Haifa, Israel. All experiments were performed according to the regulations of the Inspection Committee on the Constitution of the Animal Experimentation of the Technion-Israel Institute of Technology in Haifa, Israel from which authorization for performing animal studies was approved (Authorization No. IL-0840521). Experiments conformed to the regulations in the Prevention of Cruelty to Animals Law (Experiments on Animals) 5754–1994 and the Prevention of Cruelty to Animals Rules (Experiments on Animals) 5761–2001 Correct as of December 1, 2005.

### Isolation of immune cells from blood

Peripheral blood samples of healthy volunteers were collected in EDTA-containing tubes. Neutrophils were isolated from blood samples by negative magnetic selection with the EasySep Direct Human Neutrophil Isolation Kit (STEMCELL Technologies) according to the manufacturer’s instructions. Briefly, Isolation Cocktail (50 µl/ml) and RapidSpheres (50µl/ml) were added to a whole blood sample tube for 5 minutes. The sample tube was topped up with recommended medium and inserted into the magnet for 10 minutes. Then, sample was transferred to a new tube and RapidSpheres (50µl/ml) were added for an additional 5-minute incubation. The sample with the RapidSpheres was inserted into the magnet for 5 minutes and sample was transferred to a third tube. The last step was repeated to obtain a final clear fraction.

### Cell culture and treatment of the cells with cannabis extracts

0.2*10^6 RAW 264.7 mouse macrophages or isolated human neutrophil cells treated with 1- 2 µg/ml cannabis extract or DMSO as a control for two hours. After incubation, CBD-X treated cells were centrifuged and activated with 500 or 100 ng/ml LPS overnight, respectively. Cells were centrifuged, supernatants were collected and levels of IL-1β were detected from RAW 264.7 or IL-8, IL-6 and TNF- α levels were detected from neutrophils by ELISA with a BioTek ELISA plate reader.

### Cell viability measurement

CBD-X treated RAW 264.7 or neutrophils were washed, and medium was added to the cells with 10% Alamar Blue solution. As a negative control, Alamar Blue was added to the medium without cells. The cells were further incubated for another four hours at 37°C. The absorbance of the test and control wells was read at 570 nm and 600 nm with a BioTek ELISA plate reader.

### ELISA

One day prior to running the ELISA, Capture Antibody was diluted in 1X Coating Buffer. 100 μL of this Capture Antibody solution was added to all wells of a 96-well plate. Plates were sealed and incubate overnight (16–18 hrs.) between 2°C and 8°C. Plates were washed 4 times with at least 300 μL Wash Buffer (PBST) per well and blot residual buffer by firmly tapping plate upside down on absorbent paper. To block non-specific binding and reduce background, 200 μL 1X Assay Diluent was added per well. Plates were incubate at RT for 1 hour. Plates were washed 4 times with Wash Buffer. 100 μL/well of standards or samples were added to the appropriate wells. Plates were incubated at RT for 2 hours. Plate were washed 4 times with Wash Buffer. 11. 100 μL of diluted Detection Antibody solution was added to each well, plates were incubated at RT for 1 hour. 100 μL of diluted Avidin-HRP solution was added to each well, plates were incubated at RT for 30 minutes. Plates were washed 5 times with Wash Buffer. 100 μL of TMB Substrate was added and incubated in the dark for 10–15 minutes. Positive wells turned blue in color. Reaction was stopped by adding 100 μL of Stop Solution to each well. Positive wells should turn from blue to yellow. Absorbance was read at 450 nm and 570 nm within 15 minutes. The absorbance at 570 nm were subtracted from the absorbance at 450 nm.

### Western blot analysis for human primary isolated neutrophils

Neutrophils were isolated from healthy donors by negative magnetic selection with the EasySep Direct Human neutrophil Isolation Kit. Isolated neutrophils were activated with 100 ng/ml LPS and treated with 1 or 2 µg/ml CBD-X or DMSO as a control. After 30 minutes, the cells were collected and pelleted by centrifugation at 18800 x g and washed twice with ice-cold PBS. The washed cell pellets were resuspended in 100 µl 2x Laemmli Sample Buffer with added β-mercaptoethanol at a ratio of 1:20 (SB+βME), incubated at 100°C for 5 min and centrifuged at 18800 x g for 5 min; cell pellet containing debris was discarded. The protein samples were separated on a 10% SDS-PAGE gel and transferred onto nitrocellulose membrane using Trans-Blot Turbo Transfer Apparatus (Bio-Rad) following manufacturer’s instructions. The membrane was blocked with 5% BSA in Tris-buffered saline containing 0.1% Tween 20 followed by incubation overnight at 4°C with total or phospho-specific antibodies to P65 (1:1000) and Akt (1:1000) in 5% BSA in Tris-buffered saline containing 0.1% Tween 20. Blots were washed with Tris-buffered saline containing 0.1% Tween 20 and incubated with the respective secondary antibodies conjugated with horse radish peroxidase for one hour at room temperature. Blots were washed with Tris-buffered saline containing 0.1% Tween 20. Immunoreactive proteins were detected with the Enhanced Chemiluminescence (ECL) kit (Thermo scientific). The relative density of the protein bands was scanned using Image Quant LAS4000 and analyzed by Image-J 1.8.0-172.

### Flow cytometry

For blood samples; 10X Red Blood Cell (RBC) Lysis Buffer (BioLegend Cat. No. 420301) was diluted to 1X working concentration with DI water. 1X RBC lysis solution was added to whole blood. Samples were Incubated at room temperature for 10 minutes. samples were Centrifuged at 350xg for 5 minutes and supernatants were discarded. Samples were washed with 2% FBS in PBS by centrifugation at 350xg for 5 minutes. For blood and joint samples; fluorochrome conjugated antibodies (mentioned above) were incubated in the dark for 20 minutes. Cells were washed, resuspended in 2% FBS in PBS and 2% paraformaldehyde-PBS fixation buffer was added for flow cytometry analysis by BD LSRFortessa system.

### Collagen-induced arthritis mouse model

At day zero, C57BL/6 mice were administered with 1 mg/ml chicken collagen (200 µl/mouse) with Complete Freund Adjuvant (CFA) in the tail area, subcutaneously. At day 21, mice were injected with another boost of chicken collagen with Incomplete Freund Adjuvant (50 µl/mouse). Rheumatoid arthritis induced mice were treated with CBD-X (100 mg/Kg) every other day since day 0 (t=0), sublingually. At day 50, mice were restrained and blood was drained from the tail area and collected in EDTA tubes for down-stream analysis. RBC lysis buffer was added to the blood and centrifuged. Cells were stained with anti CD16/32 (FcR blocker), APC anti- CD45, FITC anti-CD3, BV786 anti-LY6G and BV421 anti-LY6C for flow cytometry analysis using High Throughput LSRFortessa. Alternatively, blood was centrifuged and supernatants were collected and levels of MCP-1 were detected by ELISA with a BioTek ELISA plate reader. Mice were kept for further analysis (not reported in this paper). At the end of the experiment, mice were euthanized by 5% Isoflurane and CO2.

### Collagen antibody-induced arthritis mouse model

A cocktail of four monoclonal antibodies to type II collagen (ArthritoMab; 2mg/100 µl/mouse) were injected intravenously at day 0. Mice in PBS group were injected with equal volume of sterile PBS.

At day three, all animals except PBS group were injected with LPS (100 μg/200 µl/mouse), intraperitoneal. Vehicle or CBD-X were administered twice a day, sublingually. Dexamethasone (0.5 mg/Kg) was administered every other day. At day seven, mice were anesthetized by 2% Isoflurane and blood was drained from the submandibular area and collected in EDTA tubes for blood cell count analysis using IDEXX ProCyte Dx. Then, mice were euthanized by 5% Isoflurane and CO2. Consequently, Joints were collected and disassembled by the gentleMACS™ Dissociator (3 legs, 2 ml PBS, program B then C). Fluids were filtered twice through a mesh paper or strainer during centrifugation. Supernatants were centrifuged twice and collected for ELISA. Cells were stained with anti CD16/32 (FcR blocker), APC anti- CD45, BV421 anti-CD11b, BV786 anti-LY6G and PE anti-F480 for flow cytometry analysis using High Throughput LSRFortessa.

### Statistical analysis

For statistical analysis, the ex vivo experiments data were analyzed in comparison to activated treatment as a baseline. In the case of RAW 264.7 mouse macrophages significance was determined in comparison to “LPS, DMSO” group. While in neutrophils, the significance was determined in comparison to the activated control. Data were analyzed for RAW 264.7 cells by one- way ANOVA and for neutrophils by mixed model test (Fisher’s LSD test with values p < 0.05 considered statistically significant, (*p <0.05, **p < 0.01, *** p < 0.001).

In the *in vivo* acute experiments, data for the CIA model were analyzed in comparison to collagen induced mice as a baseline named “Collagen, Vehicle”. Meanwhile, data for the CAIA model were analyzed in comparison to Arthitomab- induced mice as a baseline named “Arthitomab, Vehicle” group. Each experiment included 4–7 mice in each group. Data for the CIA model were analyzed by mixed model test and data for the CAIA model were analyzed by one-way ANOVA (Fisher’s LSD test with values p < 0.05 considered statistically significant, (*p <0.05, **p < 0.01, *** p < 0.001).

This illustration was created by https://www.biorender.com/.

## Results

### High CBD extract downregulates inflammatory cytokine secretion from macrophages and primary human neutrophils

Neutrophils migrate into the synovial fluid (SF), where they phagocytose immune complexes and release potent proteases. As key components of the innate immune system, neutrophils play a vital role in pathogenic defense. They are the first immune cells to infiltrate sites of inflammation, followed closely by monocytes (13).

Macrophages are pivotal to the pathophysiology of rheumatoid arthritis (RA). They serve as the primary source of pro-inflammatory cytokines and chemokines, such as TNF and IL-1β, and are integral to the activation of a broad spectrum of immune and non-immune cells. Furthermore, macrophages secrete various tissue-degrading enzymes, perpetuating chronic inflammation, tissue destruction, and pain responses in RA (14).

**Figure 1:**
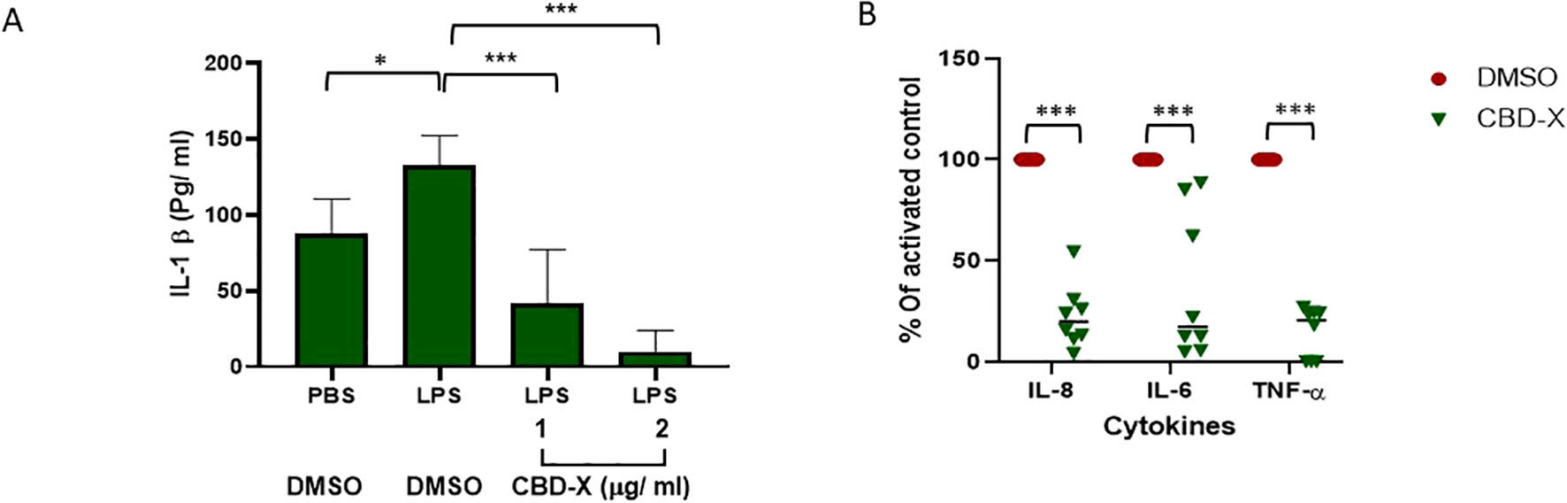
High CBD extract (CBD-X) downregulates the secretion of inflammatory cytokines from macrophages and primary human neutrophils. Mouse macrophages (RAW 264.7 cells) were treated with increasing doses of CBD-X for eight hours, Then, cells were incubated with LPS (0.5 ug/ml) for overnight and levels of I|-1 B were detected by ELISA **(A)**. Means were calculated and error bars represent the standard deviations of the means of two biologically independent experiments. Isolated human neutrophils were treated with CBD-X (2ug/ml) or DMSO as a control for two hours. Treated cells were activated by LPS (100 ng/ml) for overnight. Levels of II-8, IL-6 and TNF-a were detected by ELISA **(B)**. Means were calculated and normalized to DMSO activated cells from eight healthy donors (black line). Red dots represent DMSO treated cells and green triangles represent CBD-X treated cells. Data **(A)** were analyzed by one-way ANOVA and data **(B)** were analyzed by mixed model (Fisher's LSD test) with values of *p <0.05 and ***p<0.001 considered statistically significant.

To investigate the effects of the high-CBD extract (CBD-X) on macrophage and neutrophil activity, we first treated mouse macrophages (RAW 264.7 cells) with increasing concentrations of CBD-X. Following CBD-X treatment, the cells were stimulated with lipopolysaccharide (LPS) overnight, and the levels of IL-1β in the culture supernatant were measured using ELISA. As expected, macrophages in the LPS-stimulated DMSO control group exhibited a significant 60% increase in IL-1β secretion compared to untreated controls. In contrast, CBD-X treatment resulted in a marked, dose-dependent reduction in IL-1β levels (**Figure 1A**). To further explore this phenomenon, isolated human neutrophils from healthy donors were treated with CBD-X. The secretion levels of the pro-inflammatory cytokines IL-8, IL-6, and TNF-α were measured following activation. Notably, CBD-X treatment led to a significant reduction in cytokine secretion levels, with decreases of 75%, 76%, and 75% observed for IL-8, IL-6, and TNF-α, respectively (**Figure 1B**). To rule out the possibility that the observed effects were due to cytotoxicity caused by the CBD-X extract, cell viability was assessed using the Alamar blue assay. The results confirmed that cell viability remained unaffected by CBD-X treatment, supporting the conclusion that the observed reduction in cytokine secretion was attributable to the regulatory effects of CBD-X rather than toxicity (**Supplementary Figures S1A, B**). These findings suggest that CBD-X extract impairs the functional activity of macrophages and neutrophils, potentially modulating cell-mediated immune responses in the context of rheumatoid arthritis.

### CBD-X modulates the inflammatory signaling cascade downstream of LPS activation in primary human neutrophils

Lipopolysaccharide (LPS) activates the Toll-like receptor (TLR)-4 sensor on the cell membrane triggering myeloid differentiation primary response 88 (MYD88)-dependent and independent pathways and subsequent activation of the nuclear factor kappa B (NF-κB) or interferon regulatory factor (IRF) transcription factors (15). NF-κB induces the expression of various pro-inflammatory genes, including those encoding cytokines and chemokines, and also participates in inflammasome regulation. In addition, NF-κB plays a critical role in regulating the survival, activation, differentiation and migration of innate immune cells (16). Akt is a serine/threonine kinase that plays a crucial role in various cellular processes, including cell survival, growth, and metabolism. Akt can activate NF-κB through a few pathways, including the stimulation of IKK (IKK kinase) and p38, which ultimately leads to p65 phosphorylation and activation (17).

**Figure 2:**
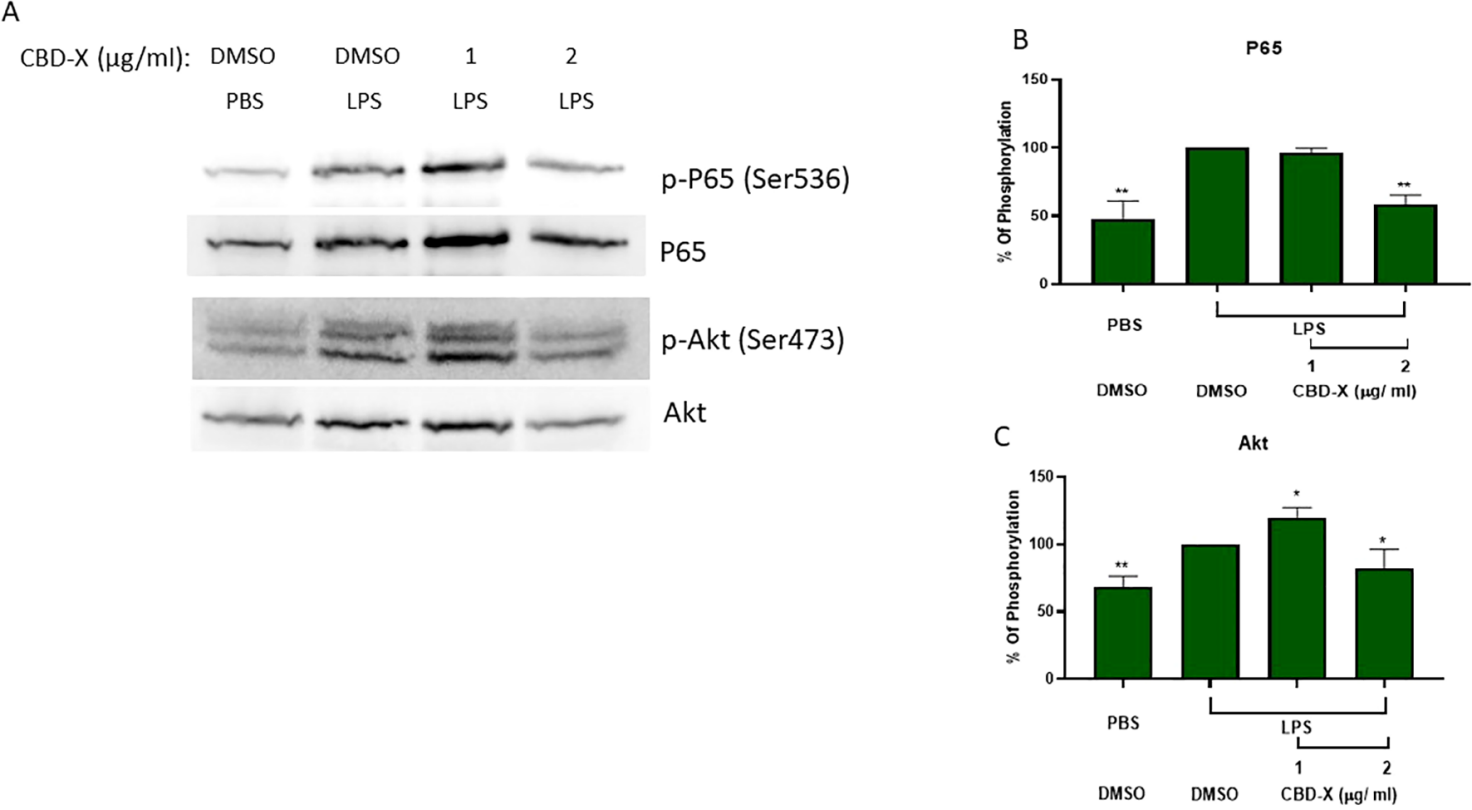
CBD-X extract modulates inflammation signaling in human derived neutrophil cells. Neutrophils were isolated from healthy donors by negative magnetic selection with the EasySep Direct Human neutrophil Isolation Kit. Isolated neutrophils were activated with 100 ng/ ml LPS and treated with 1 or 2 ug/ml CBD-X extract or DMSO as a control. After 30 minutes, the cells were collected and lysed with 100 ul 2x SB+BME. Cell lysates were analyzed for the presence of phosphorylated p 65 and Akt by western blot analysis using p$536-P65, total P65, pS473-Akt and total Akt antibodies **(A)**. Induction of phosphorylation was quantified relative to the specific total protein expression of P65 **(B)** and AKT **(C)**, Error bars represent the standard deviation of the means of two different donors, and they are expressed as average ‡ standard deviation (SD). SD were calculated in ratio to LPS activated neutrophils and data were analyzed by one-way ANOVA (Fisher's LSD test with values p < 0.05 considered statistically significant. (*p < 0.05 and **p < 0.01).

To examine the mechanism underlie the inhibitory effect of CBD-X on cytokine secretion, primary human neutrophils were isolated, activated and treated with dose dependent manner of CBD-X. Phosphorylation of P65 and Akt proteins from NF-κB signaling pathway were tested by western blot (**Figure 2A**). LPS increased the phosphorylation of P65 and Akt proteins by 50% and 25%, respectively (**Figures 2B, C**). CBD-X treatment resulted in a dose-dependent reduction in P65 phosphorylation and restored Akt phosphorylation to baseline levels at a concentration of 2 µg/mL. These findings shed light on the underlying mechanism of CBD-X’s inhibitory effects, demonstrating its ability to downregulate phosphorylation within the NF-κB signaling pathway, specifically targeting key proteins such as P65 and Akt.

### CBD-X reduces inflammatory cellular response and MCP-1 level in a collagen-induced arthritis mouse model

Rheumatoid arthritis (RA) is a chronic autoimmune and inflammatory disease characterized by alterations in multiple immune cell populations (18). Our previous studies demonstrated that CBD-X exhibits anti-inflammatory properties in murine models of lung inflammation and asthma, effectively modulating immune cell counts in lung fluids (11, 12).

**Figure 3:**
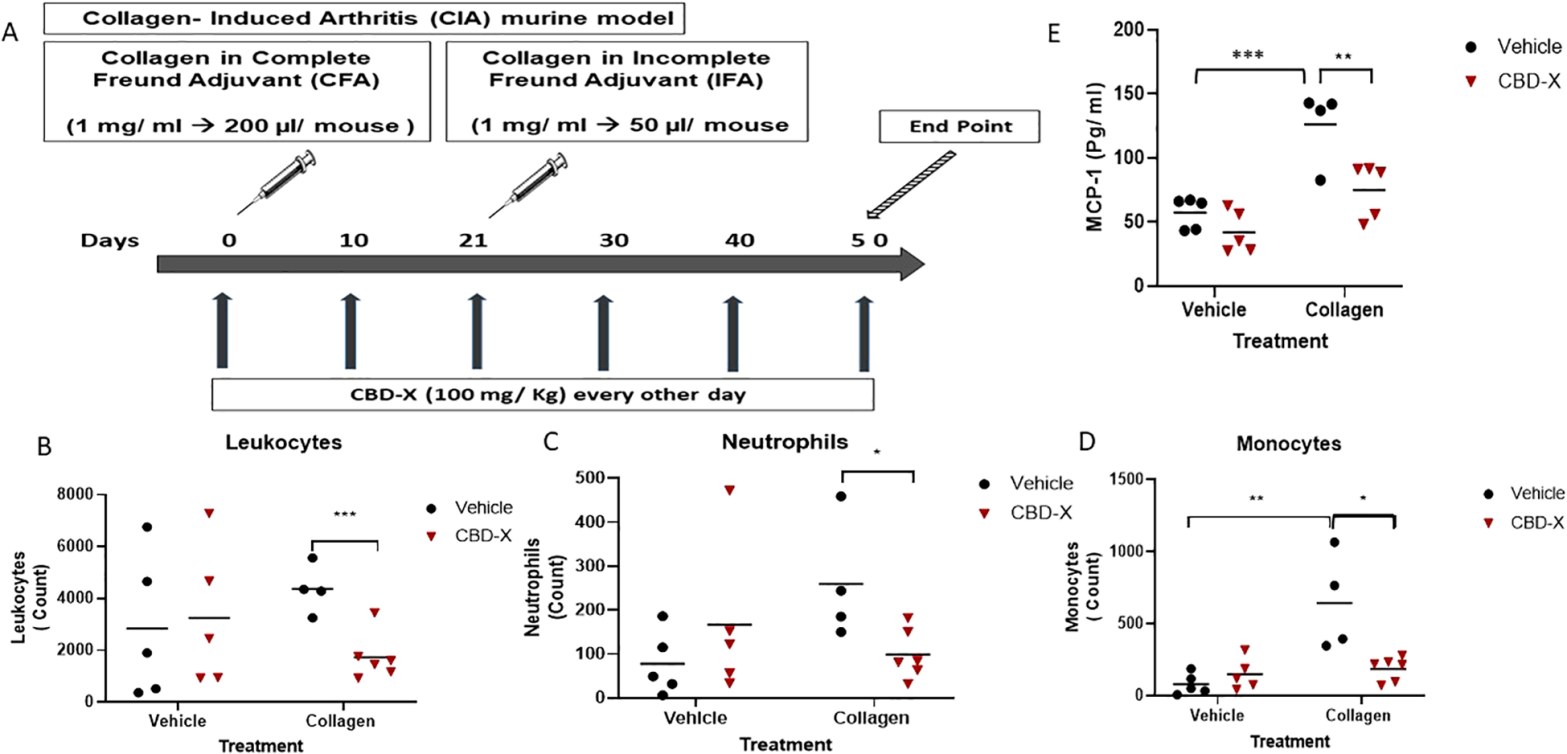
CBD-X hampers inflammatory markers in the blood of collagen-induced arthritis model. C57BL/6 mice were injected with 1 mg/ ml chicken collagen (200 l/ mouse) with complete Freund adjuvant **(A)**. At 21, mice were injected with another boost of collagen with incomplete Freund adjuvant (50 ul/ mouse). Rheumatoid arthritis induced mice were treated with CBD-X ( 100 mg/Kg) every other day since day 0, sublingu A month after the boost (day 50), blood was drained from the tail area and collected in EDTA tubes for down-stream analysis. Count of leukocytes **(B)**, neutrophils **(C)** and monocytes **(D)** were detected in the blood by cytometry. MCP-1 pro-inflammatory cytokine levels were detected by ELISA **(E)**. Means were calculated in each group (black line). mice were divided for four treatment groups; vehicle (n= 5), CBD-X (n=5), Collagen- ve (n=4) and Collagen-CBD-X (n=6). Black dots represent the vehicle treated group and red triangles represent the CBD-X treated group. Each shape (dot or triangle) represents one case and results were analyzed by m model (Fisher's LSD test) with values of *p <0.05, **p<0.01, ***p<0.001 considered statistically significant.

To elucidate the cellular effects of CBD-X on autoimmune arthritis, we established a collagen-induced arthritis (CIA) murine model and initiated treatment with CBD-X from day zero of immunization (**Figure 3A**). Vehicle-treated mice were served as a negative control group. CBD-X treatment in collagen-induced mice reduced levels of leukocyte, neutrophil and monocyte by 60%, 56% and 64%, respectively (**Figures 3B–D**). Whereas no such effect was observed in vehicle-treated mice. The gating strategy for flow cytometry analysis are shown (**Supplementary Figures S4A–SD**). Flow cytometry raw data are provided (**Supplementary Figures S5**–**S8**).

MCP-1, a chemotactic cytokine highly expressed in the serum, synovial fluid, and tissues of RA patients, plays a critical role in recruiting monocytes, macrophages, and lymphocytes to sites of inflammation (19).

Consistent with its anti-inflammatory properties, CBD-X treatment of collagen-induced mice reduced MCP-1 levels in the blood by twofold (**Figure 3E**), while showing no effect in vehicle-treated mice.Similarly, the therapeutic treatment protocol was initiated 21 days after rheumatoid arthritis onset (**Supplementary Figure S3**). At this stage of disease progression, CBD-X treatment led to a marked reduction in leukocyte, neutrophil, and monocyte counts in the blood (**Supplementary Figures S3B–SD**). In contrast, no significant changes in these immune cell populations were observed in healthy mice treated with the same concentration of CBD-X (**Supplementary Figure S2**). Additionally, levels of the inflammatory cytokines MCP-1 and IL-6 remained unchanged, and body weight was stable throughout the four-week monitoring period (**Supplementary Figure S2**). These findings indicate that the CBD-X dosage effective in RA models does not elicit measurable immunological or physiological side effects in healthy mice, supporting its favorable safety profile under non-inflammatory conditions.

Collectively, these findings highlight CBD-X’s specific action in inflammatory conditions associated with autoimmune arthritis and support its therapeutic potential in autoimmune inflammation, while underscoring its favorable safety profile under non-inflammatory conditions.

### CBD-X reduces inflammatory cell levels in the blood of collagen antibody-induced arthritis mouse model

The antibody-induced arthritis model offers several advantages over the classic collagen-induced arthritis (CIA) model, including rapid disease onset, greater synchronicity, and a shorter protocol duration of 1–2 weeks (20).

Building on this framework, we sought to investigate whether CBD-X could mitigate inflammation in an antibody-induced arthritis mouse model of rheumatoid arthritis and reduce immune cell concentrations in the bloodstream.

**Figure 4:**
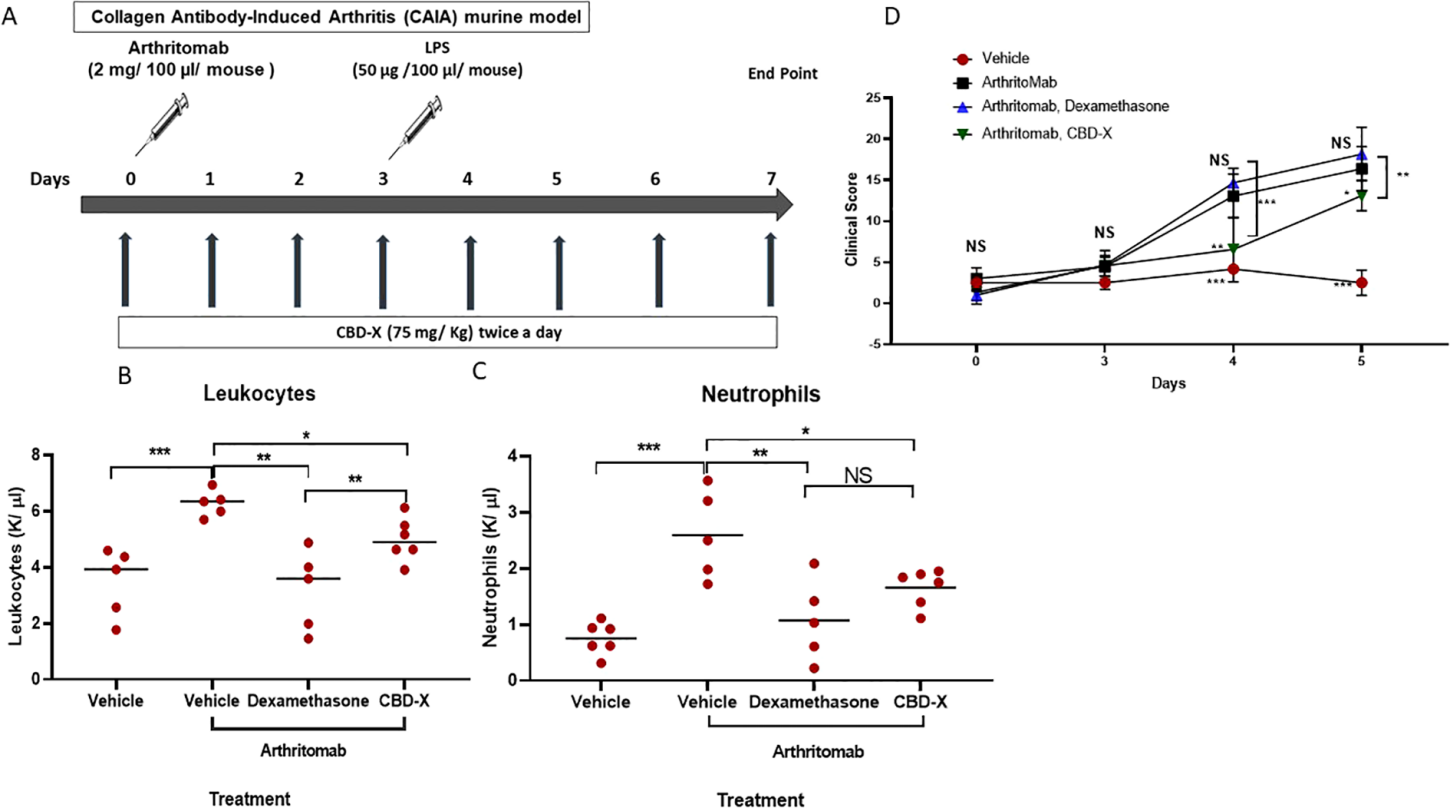
CBD-X hampers inflammatory cells in the blood of murine rheumatoid arthritis model. C57BL/6 mice were injected with ArthritoMab (2 mg/ 100 pl/ mouse; a cocktail of four monoclonal antibodies to type Il collagen). At day three, mice were injected with LPS (50 ug /100 ul/ mouse). Rheumatoid arthritis induced mice were treated with CBD-X (75 mg/ Kg) twice a day or Dexamethasone every other day, sublingually. Mice were sacrificed at day seven, blood was drained from the submandibular area and collected in EDTA tubes for cell count analysis **(A)**. Concentrations of leukocytes **(B)**, and neutrophils **(C)** were detected in the blood. Clinical score is shown **(D)**. mice were divided for four treatment groups; vehicle (n= 5), ArthritoMab (n=5), ArthritoMab- Dexamethasone (n=5) and ArthritoMab-CBD-X (n=6). Means were calculated in each group (black line) and each red dot represents one case. Results were analyzed by one way ANOVA (Fisher's LSD test) with values of *p <0.05, **p<0.01, ***p<0.001 considered statistically significant. NS is considered not statistically significant.

To achieve this, we utilized the collagen antibody-induced arthritis (CAIA) model, employing ArthritoMab (a monoclonal antibody cocktail) to induce rheumatoid arthritis (**Figure 4A**). Rheumatic mice were treated with Dexamethasone or the high-CBD extract (CBD-X), and blood samples were collected for analysis. Following RA induction, blood concentrations of leukocytes and neutrophils increased significantly—by approximately 1.7- and 2.5-fold, respectively (**Figures 4B, C**). Notably, treatment with either dexamethasone or CBD-X significantly reduced these elevated levels, effectively attenuating inflammatory cell infiltration in the blood. Furthermore, ArthritoMab administration led to a 5- and 6-fold increase in clinical scores on days 4 and 5, respectively (**Figure 4D**). CBD-X treatment significantly reduced these clinical scores, whereas dexamethasone did not produce a comparable effect.

These findings further support the potential of CBD-X as an anti-inflammatory therapeutic agent in murine models of rheumatoid arthritis.

### CBD-X reduces neutrophil count and neutrophil/macrophage ratio but not its Dexamethasone counterpart in the joints of collagen antibody-induced arthritis mouse model

**Figure 5:**
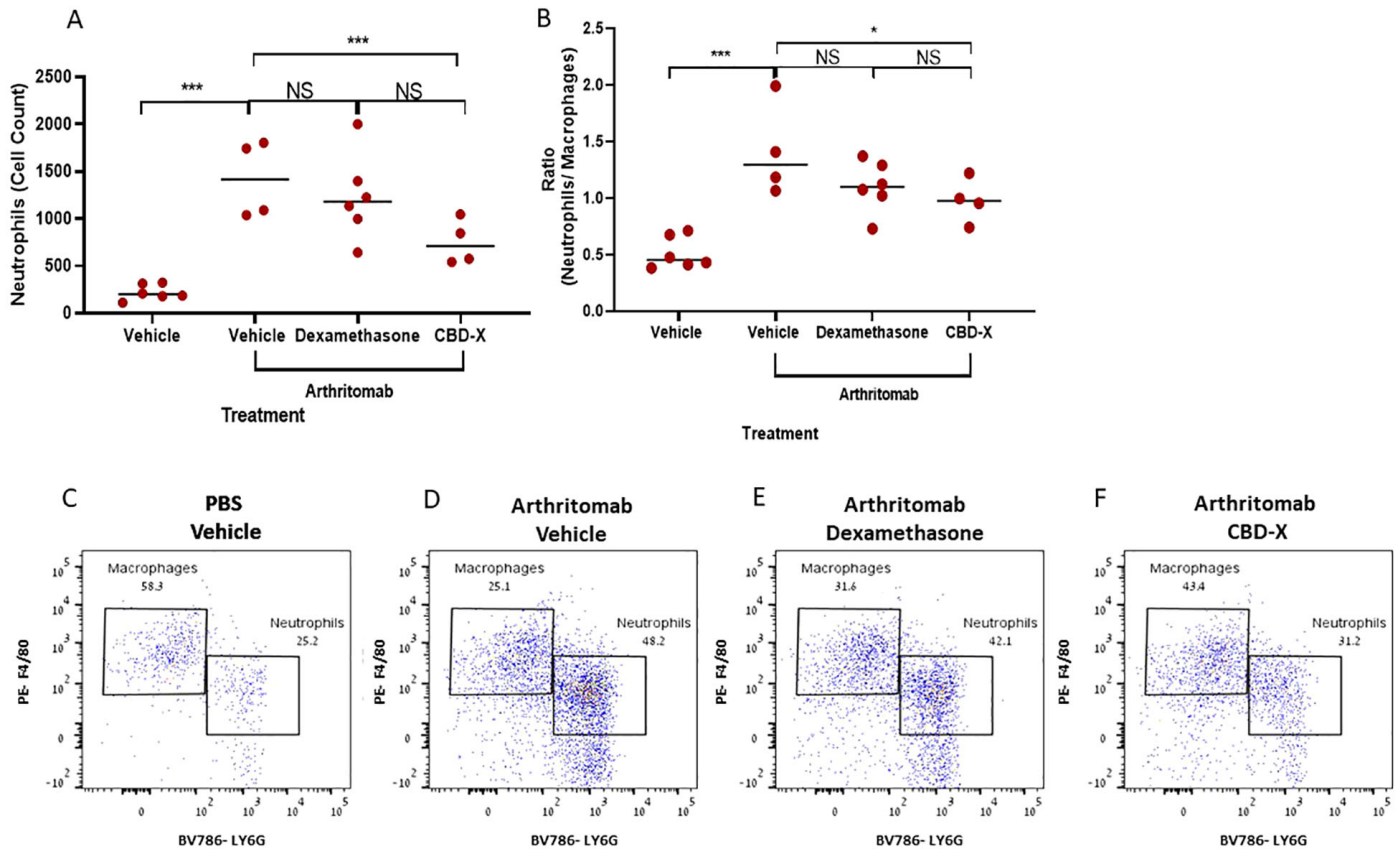
CBD-X inhibits neutrophil presence in the joints, but not Dexamethasone in a murine rheumatoid arthritis model. C57BL/6 mice were injected with ArthritoMab (2 mg/ 100 ul/ mouse; a cocktail of four monoclonal antibodies to type Il collagen). At day three, mice were injected with LPS (50 ug /100 pl/ mouse). Rheumatoid arthritis induced mice were treated with Dexamethasone (0.5 mg/ Kg) every other day or CBD-X (75 mg/ Kg) twice a day, sublingually. Mice were sacrificed at day seven, joints were homogenized, filtered and centrifuged. Cells were stained with anti CD16/32 (FcR blocker), APC anti-CD45, BV421 anti-CD11b, BV786 anti-LY6G and PE anti-F480 for flow cytometry analysis. Cell count of neutrophils **(A)** were detected and neutrophil/ macrophage ratio was calculated **(B)**. Dot plots for BV786- LY6G vs PE-F4/80 gate were shown for the different treatments; PBS- vehicle (n=6), Arthritomab-vehicle (n=4), Arthritomab-Dexamethasone (n=6) and Arthritomab-CBD-X (n=4, **C–F**). Means were calculated in each group (black line) and each red dot represents one case. Results were analyzed in comparison to Arthritomab- Vehicle group by one way ANOVA (Fisher's LSD test) with values of *p <0.05 and ***p<0.001 considered statistically significant. NS considered non significant.

Neutrophils exhibit an activated phenotype in the peripheral blood, and activated neutrophils are found in high numbers within the synovial joints and tissues of patients with rheumatoid arthritis (21). Furthermore, the neutrophil-to-monocyte ratio has been identified as a novel inflammatory marker associated with rheumatoid arthritis activity (22). Adding to that, Dexamethasone therapy in RA patients leads to a rapid, clinically beneficial effect (23). To evaluate the therapeutic potential of CBD-X, we established a murine model of rheumatoid arthritis and treated the mice with CBD-X. Rheumatic mice were sacrificed; their joints were isolated, homogenized, and filtered. The resulting joint cells were centrifuged and analyzed via flow cytometry. Dexamethasone served as the corticosteroid therapy for rheumatoid arthritis treatment control. Induction of rheumatoid arthritis using ArthritoMab significantly elevated neutrophil levels by 6.2-fold (**Figure 5A**) and increased the neutrophil-to-macrophage ratio by 2.7-fold (**Figure 5B**). Treatment with high doses of CBD-X significantly reduced these elevations, whereas Dexamethasone did not. Representative dot plots from the flow cytometry analysis illustrate macrophage and neutrophil populations across the treatment groups (**Figures 5C–F**). The gating strategy for flow cytometry analysis are shown (**Supplementary Figures S9A–D**). Flow cytometry raw data are provided (**Supplementary Figures S10**–**S13**).

These findings underscore the inhibitory effect of CBD-X on inflammatory cell populations in comparison to its Dexamethasone counterpart and highlight its therapeutic potential and competitive advantage in treating rheumatoid arthritis.

### CBD-X mitigates the imbalance in the ratio of CD11b- high vs CD11b-low macrophages in murine rheumatic joints

**Figure 6:**
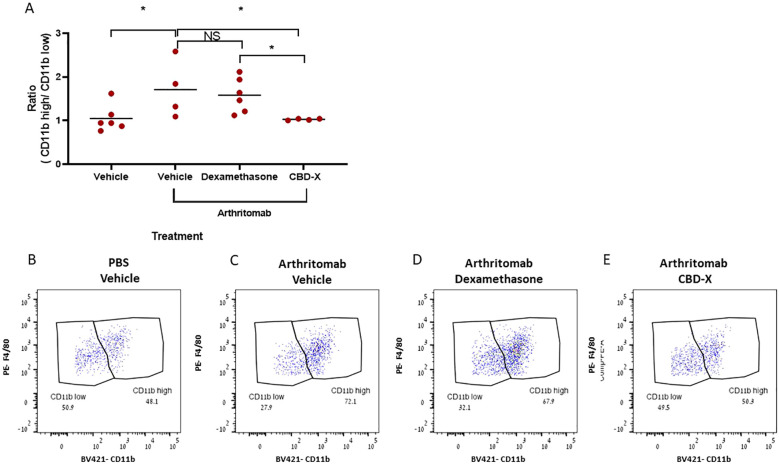
CBD-X mitigates the imbalance in the number of CD11b high vs CD11b low macrophages in murine rheumatic joints. C57BL/6 mice were injected with ArthritoMab and were treated with Dexamethasone or CBD-X, sublingually. Mice were sacrificed at day seven, joints were homogenized, filtered and centrifuged. Cells were stained with anti CD16/32 (FcR blocker), APC anti-CD45, BV421 anti-CD11b, BV786 anti-LY6G and PE anti-F480 for flow cytometry analysis. CD11b high vs CD11b low macrophages were detected from parent gate of macrophages and ratios were calculated **(A)**. Dot plots for BV421- CD11b vs PE-F4/80 gate were shown for the different treatments; PBS- vehicle (n=6), Arthritomab-vehicle (n= 4), Arthritomab-Dexamethasone (n= 6) and Arthritomab-CBD-X (n= 4, **B–E**). Means were calculated from each group (black line) and each red dot represents one case. Results were analyzed in comparison to Arthritomab- Vehicle group by one way ANOVA (Fisher's LSD test) with values of *p <0.05 considered statistically significant. NS considered non significant.

CD11b-low population of macrophages display pro-resolving properties important for completing the resolution sequel and for communicating the return to a homeostatic state at lymphoid organs during resolution of the acute inflammatory response (24). In order to characterize the macrophage phenotype in the murine rheumatic joints post CBD-X treatment, CD11b surface marker was used and analyzed by flow cytometry. Arthritomab increased the number of CD11b- high macrophages in ratio to CD11b-low population almost by 2 folds in comparison to control (**Figure 6A**). However, CBD-X attenuated this ratio back to baseline but not Dexamethasone counterpart. Representative dot plots of macrophages including CD11b-high and CD11b-low sub-populations of the different treatments are shown (**Figures 6B–E**).

Apparently, CBD-X increased the pro-resolving macrophages CD11b-low on the expense of the pro-inflammatory macrophages CD11b-high. These results support the notion that CBD-X promotes anti-inflammatory effect by mitigating the imbalance of CD11b sub-populations and maintaining the homeostasis between CD11b- high vs CD11b-low macrophages.

### CBD-X affects cytokine levels in a collagen antibody-induced arthritis mouse model

Cytokines play a central role in regulating inflammatory processes implicated in the pathogenesis of rheumatoid arthritis (RA). Within rheumatoid joints, an imbalance between pro- and anti-inflammatory cytokine activities is well-documented, promoting autoimmunity, chronic inflammation, and ultimately joint damage (2). Among these, TNF-α is a pivotal pro-inflammatory cytokine in RA, making its regulation crucial for managing the disease. IL-10, a pleiotropic cytokine, is considered a promising modulator for controlling RA (25). Furthermore, monocyte chemoattractant protein 1 (MCP-1) is highly expressed in the joints of patients with RA (19).

To investigate the effect of CBD-X extract on cytokine levels, we examined its impact on blood and joint cytokines in a collagen antibody-induced arthritis (CAIA) murine model of rheumatoid arthritis.

**Figure 7:**
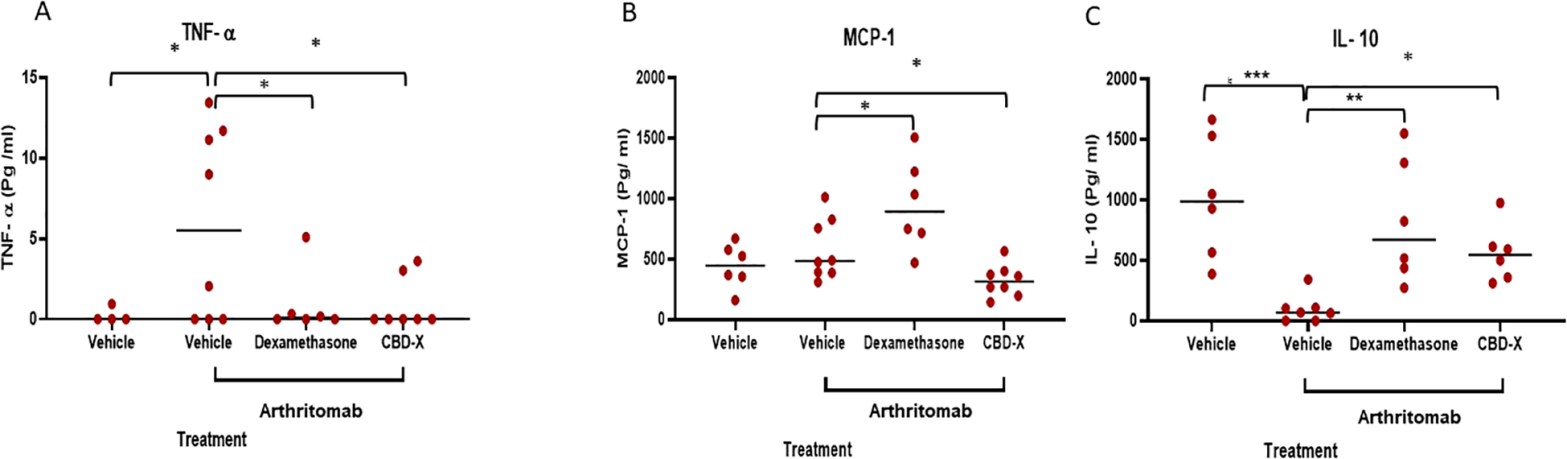
High CBD extract (CBD-X) promotes immune-reprogramming in a murine rheumatoid arthritis model . C57BL/6 mice were injected with ArthritoMab (2 mg/ 100 ul/ mouse; a cocktail of four monoclonal antibodies to type Il collagen). At day three, mice were injected with LPS (50 g/100 pl/ mouse). Rheumatoid arthritis induced mice were treated with Dexamethasone (0.5 mg/ Kg) every other day or with CBD-* (75 mg/Kg) twice a day, in a sub lingual manner. Mice were sacrificed at day seven, blood was drained and joints were homogenized and filtered. Levels of TNF- a from the blood **(A)** were detected by ELISA. Also, MCP-1 and IL-10 **(B, C)** levels in the joint interstitial fluids were detected by ELISA. mice were divided for four treatment groups; vehicle (TNF- a n= 4, MCP-1 and IL-10 n= 6), ArthritoMab (n=8), ArthritoMab-Dexamethasone (n=6) and ArthritoMab-CBD-X (TNF- a n= 7, MCP-1 n= 8, IL-10 n=6). Averages were calculated in each group and results were analyzed in comparison to Arthritomab-Vehicle group by one way ANOVA (Fisher's LSD test) with values of *p <0.05, **p<0.01, ***p<0.001 considered statistically significant.

Our findings revealed that ArthritoMab-induced rheumatoid arthritis resulted in significantly elevated levels of the pro-inflammatory cytokines TNF-α in the blood but not MCP-1 in the joints. However, treatment with CBD-X reduced TNF-α and MCP-1 levels by 84% and 55%, respectively, bringing them back to baseline levels (**Figures 7A, B**). While Dexamethasone effectively reduced TNF-α levels, it failed to decrease MCP-1 levels. Conversely, ArthritoMab-induced mice exhibited a 68% reduction in the anti-inflammatory cytokine IL-10 compared to the vehicle control (**Figure 7C**). Treatment with CBD-X significantly elevated IL-10 levels, similar to the effect observed with Dexamethasone. Notably, IL-6 cytokine level in the blood and IL-1β level in the joints show no significant changes (**Supplementary Figures S14A, B**). In addition, TNF-α level in the joints were not detectable.

These results demonstrate that CBD-X treatment exerts an anti-inflammatory effect by reducing pro-inflammatory cytokines also enhances anti-inflammatory IL-10 levels highlighting its therapeutic potential in RA management.

## Discussion

RA is a typical chronic disease, in which the failure of spontaneous resolution of inflammation causes the disease to persist (3).

Several genetic and environmental (microbiota, smoking, infectious agents) factors contribute to its pathogenesis. Although convention treatment strategies, predominantly Disease Modifying Anti Rheumatic Drugs (DMARDs) and Glucocorticoids (GC), are unchanged as the primary line of treatment. Novel strategies consisting of biological DMARDs, are being developed and explored. Personalized approaches using biologicals target specific pathways associated with disease progression (26).

However, current treatments for rheumatoid arthritis are associated with substantial economic burden and undesirable side effects, underscoring the urgent need for innovative therapeutic approaches. In this study, we demonstrated the significant therapeutic potential of the CBD-X extract, derived from a high-CBD cannabis strain, in managing rheumatoid arthritis. Previous studies have shown that cannabis can inhibit the proliferation of synovial fibroblasts, suppress the secretion of pro-inflammatory cytokines by immune cells, and reduce the production of nitric oxide synthases, including inducible NO synthase (iNOS), in chondrocytes, thereby preventing cartilage damage (8).

Furthermore, our prior research demonstrated the beneficial effects of the CBD-X extract in modulating cytokine storms and improving outcomes in acute lung disease and asthma (7, 8, 11, 12). These findings motivated us to explore its efficacy in a rheumatoid arthritis disease model.

To effectively regulate the immune response in chronic inflammatory diseases like rheumatoid arthritis, it is essential to counteract the sustained activation of innate immune effector cells, such as neutrophils and monocytes, which perpetuate inflammation within the synovial membrane (3). When macrophages or neutrophils encounter inflammatory stimuli, they secrete pro-inflammatory cytokines, which further drive the inflammatory response (27).

In this study, we initially evaluated the effect of the CBD-X extract on the mouse macrophage cell line RAW 264.7, focusing on its ability to inhibit the production of the pro-inflammatory cytokine IL-1β. Additionally, we investigated the impact of CBD-X on primary human neutrophils, the first responders of innate immunity, and their secretion of pro-inflammatory cytokines. Notably, our findings demonstrated that CBD-X extract significantly reduced cytokine secretion in both cell types. Specifically, it suppressed IL-1β production in activated macrophages and inhibited the secretion of IL-8, IL-6, and TNF-α in activated primary human neutrophils.

Consequently, to elucidate the mechanism underlie the inhibitory effect of CBD-X, primary neutrophils were activated, treated with CBD-X and protein phosphorylation down-stream were examined. Akt can activate NF-κB through a few pathways, including the stimulation of IKK (IKK kinase) and p38, which ultimately leads to p65 phosphorylation and activation (17). CBD-X decreased the phosphorylation of LPS-induced signaling pathway proteins including P65 and Akt. Dysregulation of the NF-κB pathway contributes to the inhibitory effect of CBD-X and alleviates inflammatory diseases like rheumatoid arthritis.

To assess the therapeutic potential of CBD-X *in vivo*, we conducted studies using a rheumatoid arthritis mouse model. Initially, we employed a collagen-induced arthritis model to measure leukocyte, neutrophil and monocyte counts, as well as the levels of the inflammatory cytokine MCP-1 in the blood. Treatment with CBD-X significantly reduced all these parameters in diseased mice, while no effects were observed in healthy, non-diseased controls. Moreover, both approaches; preventive and therapeutic of CBD-X administration to rheumatic mice were inhibitory. This finding underscores the regulatory specificity of CBD-X, indicating its action is limited to conditions of disturbed homeostasis, such as inflammation, which is critical for its future clinical application.

To further examine our findings in an alternative rheumatoid arthritis model, we utilized a collagen antibody-induced arthritis model, which involves the administration of ArthritoMab—a cocktail of four arthritogenic monoclonal antibodies targeting collagen II (CII). CBD-X treatment in this model resulted in a marked reduction in leukocyte, neutrophil, and monocyte levels in the blood, as well as a significant decrease in neutrophil infiltration within the joints. These results further support the potential of CBD-X as an effective therapeutic agent for rheumatoid arthritis.

As an additional measure, we investigated the neutrophil–monocyte ratio (NMR), a novel inflammatory hematological marker. Studies have shown that NMR correlates with rheumatoid arthritis disease activity and aligns with classical markers such as C-reactive protein (CRP), erythrocyte sedimentation rate (ESR), and rheumatoid factor (RF) (22). Consequently, we were intrigued to calculate the NMR in the joints of CBD-X-treated mice. The results revealed a significant reduction in NMR levels compared to ArthritoMab-induced controls, highlighting the anti-inflammatory impact of CBD-X. Notably, Dexamethasone considered part of the corticosteroid therapy and has immunosuppressive effect and leads to a rapid, clinically beneficial effect in RA patients (23). Notably, CBD has a similar anti-inflammatory effect to Dexamethasone by attenuating the LPS-induced production of NO, IL-6, and TNF-α (28). However, only CBD attenuated JNK phosphorylation levels, and only DEX attenuated IKK phosphorylation levels (28). Importantly, CBD-X showed a competitive advantage to Dexamethasone counterpart in reducing clinical score, neutrophil infiltration to the joints and consequently NMR reduction levels.

Moreover, CD11b-low macrophages display pro-resolving properties; poorly responsive to activation in terms of cytokine and chemokine secretion, loss of phagocytic potential and are prone to migrate to lymphoid organs (24). CBD-X Promotes its anti-inflammatory effect by recruiting pro-resolving macrophages CD11b-low on the expense of the pro-inflammatory macrophages CD11b-high.

Rheumatoid arthritis patients are characterized by elevated levels of pro-inflammatory cytokines such as TNF-α, IL-6, IL-1β, and CRP, which reflect heightened inflammatory activity. Simultaneously, decreased levels of the anti-inflammatory cytokine IL-10 may contribute to disease exacerbation (29). To determine whether CBD-X extract influences cytokine release in the rheumatoid arthritis mouse model, we measured its effects on key inflammatory mediators. Our findings demonstrated that CBD-X exerts anti-inflammatory effects by inhibiting TNF-α and MCP-1also promotes upregulating anti-inflammatory IL-10 cytokine levels.

**Figure 8:**
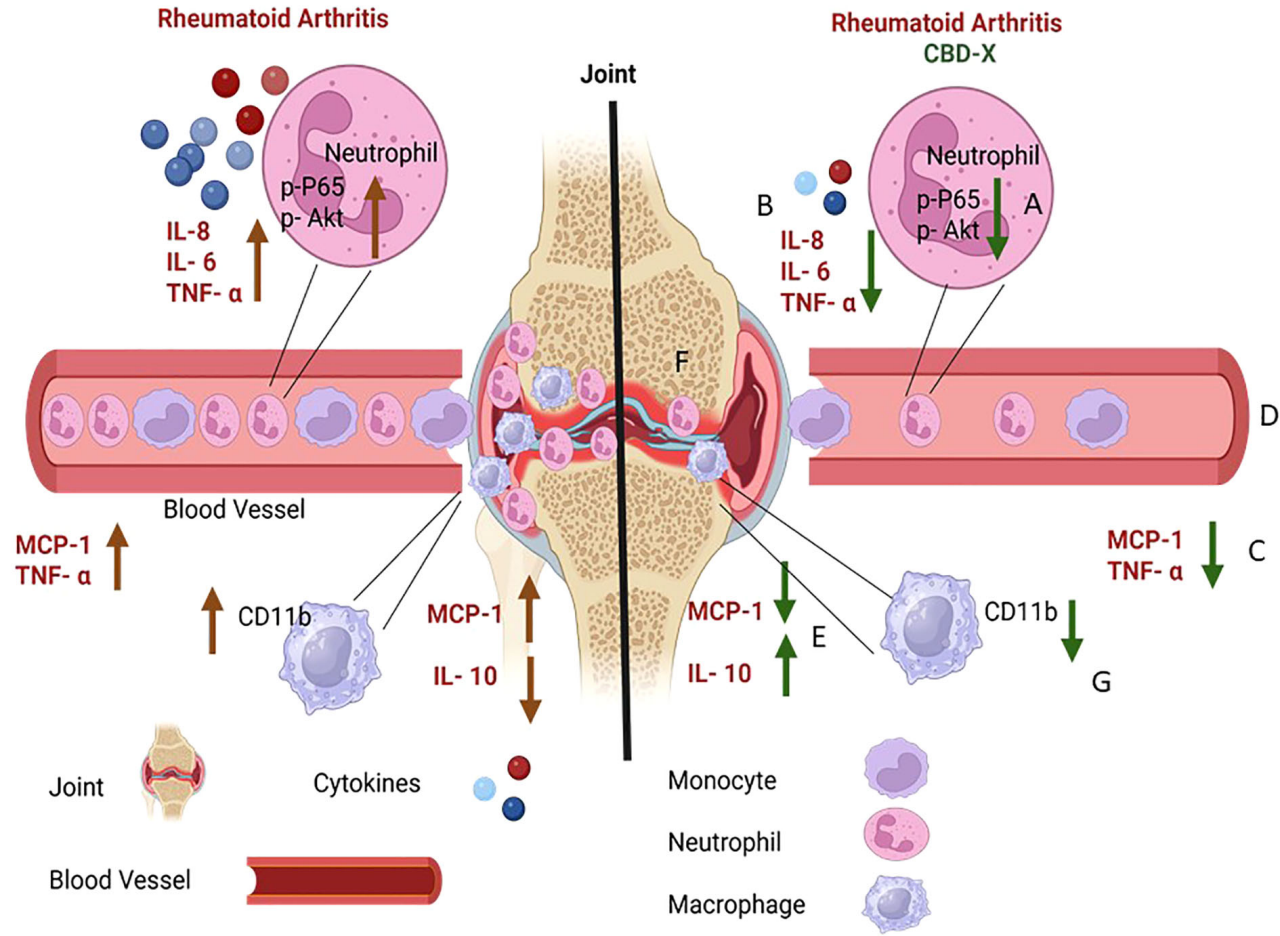
Illustration for CBD-X effect on immune cells and cytokine secretion in the inflamed joint of RA model. High CBD extract named CBD-X hampers P65 and Akt phosphorylation in neutrophils **(A)**. CBD-X attenuates the activation of neutrophils expressed by reducing the levels of IL-8, IL-6 and TN- a **(B)**. Furthermore, CBD-X inhibits pro-inflammatory cytokines MCP-1 and TNF-a in the blood **(C)**. In addition, CBD-x reduces leukocyte levels in the blood stream **(D)**. Importantly, CBD-X inhibits the pro-inflammatory cytokine MCP-1 and elevates the anti- inflammatory cytokine IL- 10 in the joints **(E)** Consequently, CBD-X suppresses the immune cell infiltration to the joints **(F)**. Notably, CBD-X promotes pro-resolving CD1b- low macrophages recruitment to the joints **(G)**. To sum up, CBD-X has anti- inflammatory effect and has therapeutic advantage on RA disease. This Illustration was created by https://www.biorender.com/.

In this study, we explored the therapeutic potential of high CBD extract termed CBD-X by determining its effects on cells involved in the rheumatoid arthritis disease. We established two ex vivo cell models: mouse macrophages and primary human neutrophils to examine the anti-inflammatory properties of CBD-X. Our research results reveal that CBD-X extract inhibits the secretion of pro-inflammatory cytokines IL-1β from macrophages and IL-8, IL-6 and TNF-α from neutrophils. Moreover, in murine models of rheumatoid arthritis, CBD-X administration significantly downregulated leukocyte levels in the blood. This reduction was primarily attributed to a decrease in blood leukocytesin both models; collagen-induced arthritis (CIA) and collagen antibody-induced arthritis (CAIA). Furthermore, CBD-X extract reduced neutrophil migration to the joints in the CAIA model. Notably, CBD-X treatment also affected the neutrophil-to-macrophage ratio (NMR), an important marker of rheumatoid arthritis, further supporting its regulatory effects on inflammatory cell populations. Importantly, CBD-X promotes the pro-resolving CD1b-low macrophages recruitment to the inflamed site. Lastly, CBD-X promotes inhibitory effect in the rheumatoid arthritis diseased model. This expressed by downregulating the pro-inflammatory cytokines TNF-α and MCP-1 and upregulating the anti-inflammatory IL-10 (illustrated in **Figure 8**). These results pave the way for further clinical studies to validate the efficacy and safety of CBD-X extract as a potential treatment for the chronic inflammatory disease rheumatoid arthritis.

## Data Availability

The original contributions presented in the study are included in the article/**Supplementary Material**. Further inquiries can be directed to the corresponding author.
