# Supplementary material for: High CBD extract (CBD-X) modulates inflammation and immune cell activity in rheumatoid arthritis
Source: Front Immunol. 2025 Jul 10;16:1599109. doi: 10.3389/fimmu.2025.1599109 (PMC12286807; doi:10.3389/fimmu.2025.1599109)
Supplement: Supplementary file 1 [file Presentation1.pptx]

## Slide 1
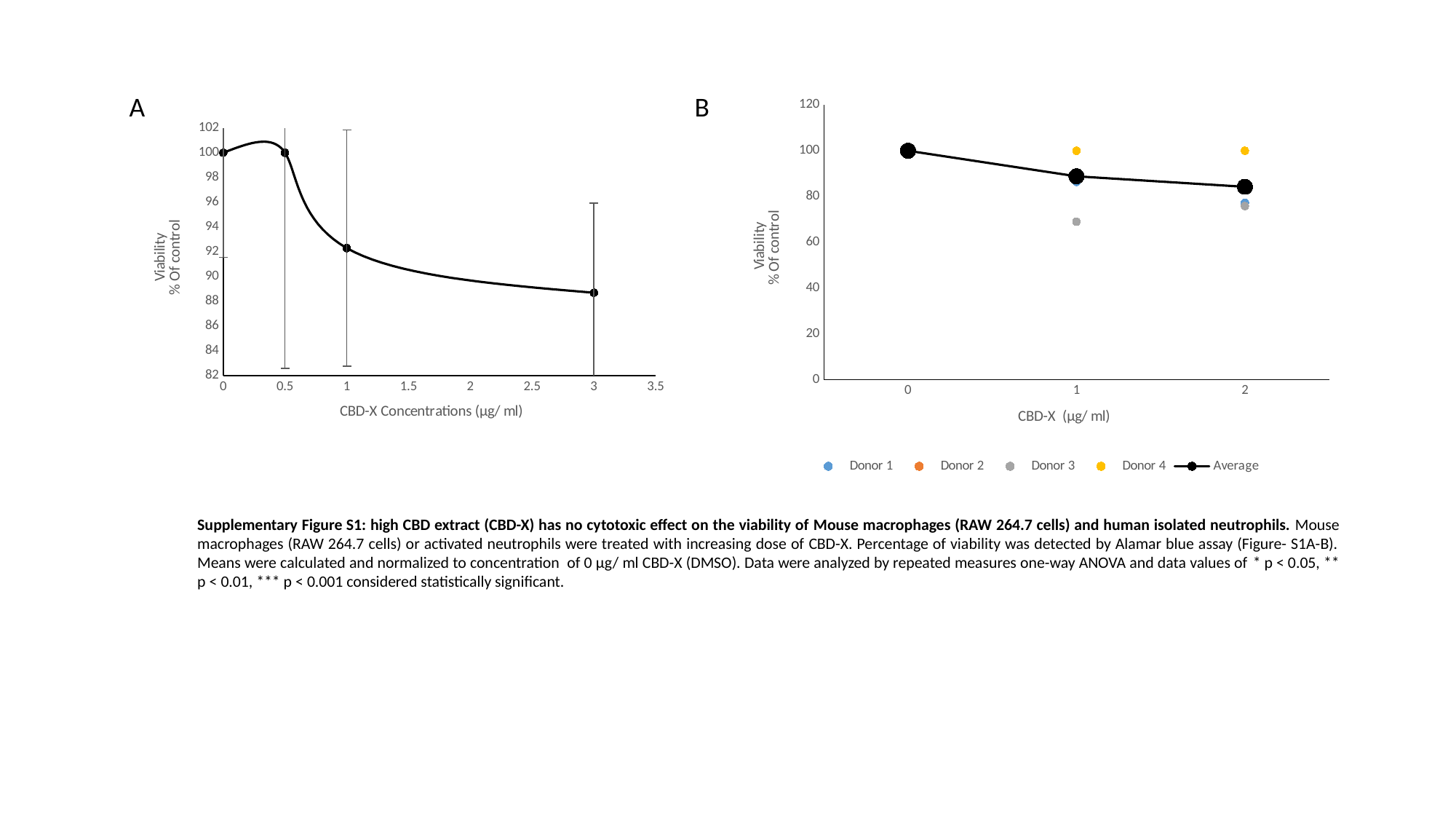

A
B
### Chart
| Category | Donor 1 | Donor 2 | Donor 3 | Donor 4 | Average |
|---|---|---|---|---|---|
| 0 | 100.0 | 100.0 | 100.0 | 100.0 | 100.0 |
| 1 | 86.35725565888752 | 100.0 | 68.99379432624113 | 100.0 | 88.83776249628217 |
| 2 | 77.22407439901737 | 83.94363496932516 | 75.81117021276596 | 100.0 | 84.24471989527711 |
### Chart
| Category | % Of viability |
|---|---|Supplementary Figure S1: high CBD extract (CBD-X) has no cytotoxic effect on the viability of Mouse macrophages (RAW 264.7 cells) and human isolated neutrophils. Mouse macrophages (RAW 264.7 cells) or activated neutrophils were treated with increasing dose of CBD-X. Percentage of viability was detected by Alamar blue assay (Figure- S1A-B). Means were calculated and normalized to concentration of 0 µg/ ml CBD-X (DMSO). Data were analyzed by repeated measures one-way ANOVA and data values of * p < 0.05, ** p < 0.01, *** p < 0.001 considered statistically significant.

## Slide 2
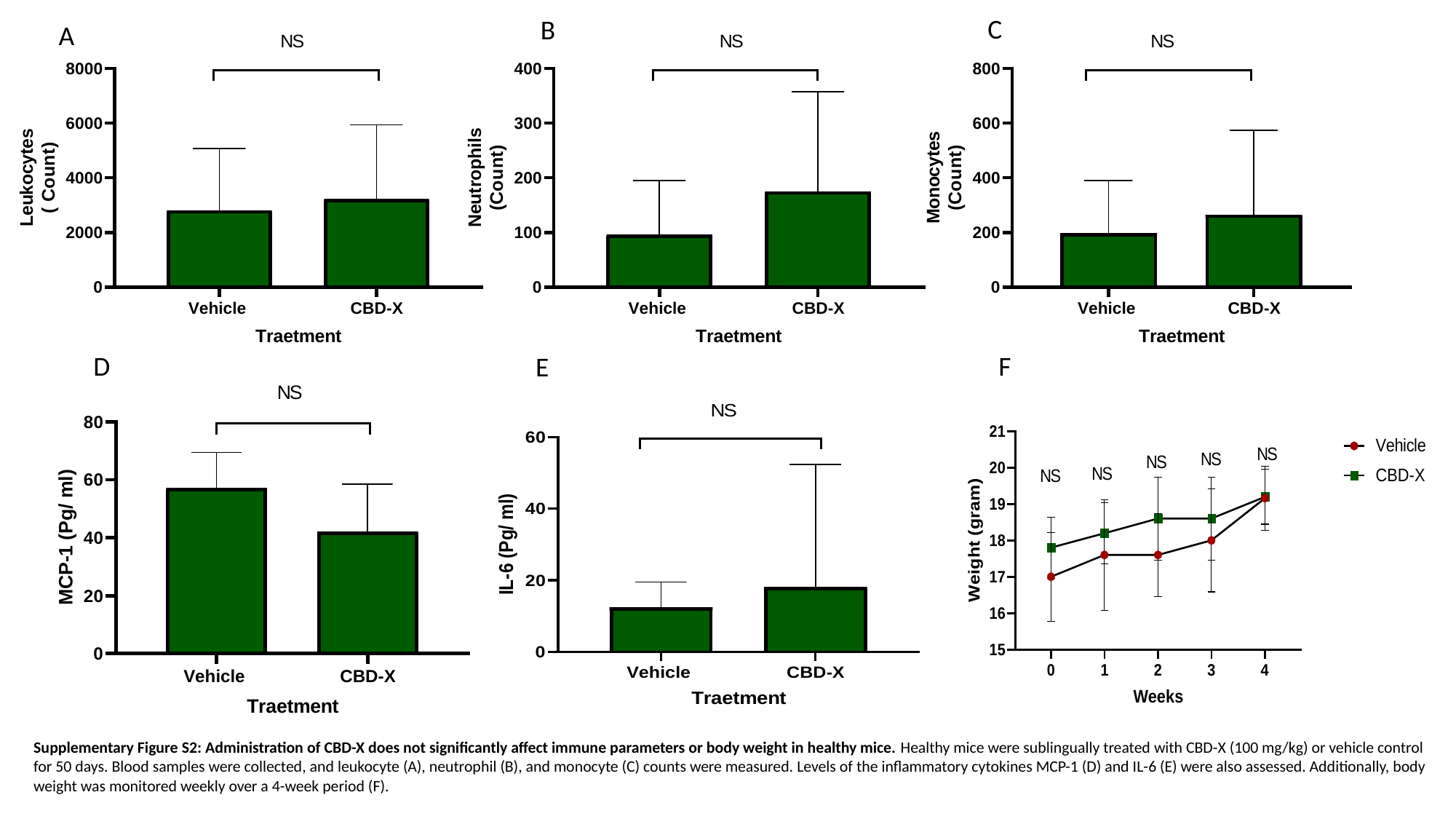

C
B
A
D
F
E
Supplementary Figure S2: Administration of CBD-X does not significantly affect immune parameters or body weight in healthy mice. Healthy mice were sublingually treated with CBD-X (100 mg/kg) or vehicle control for 50 days. Blood samples were collected, and leukocyte (A), neutrophil (B), and monocyte (C) counts were measured. Levels of the inflammatory cytokines MCP-1 (D) and IL-6 (E) were also assessed. Additionally, body weight was monitored weekly over a 4-week period (F).

## Slide 3
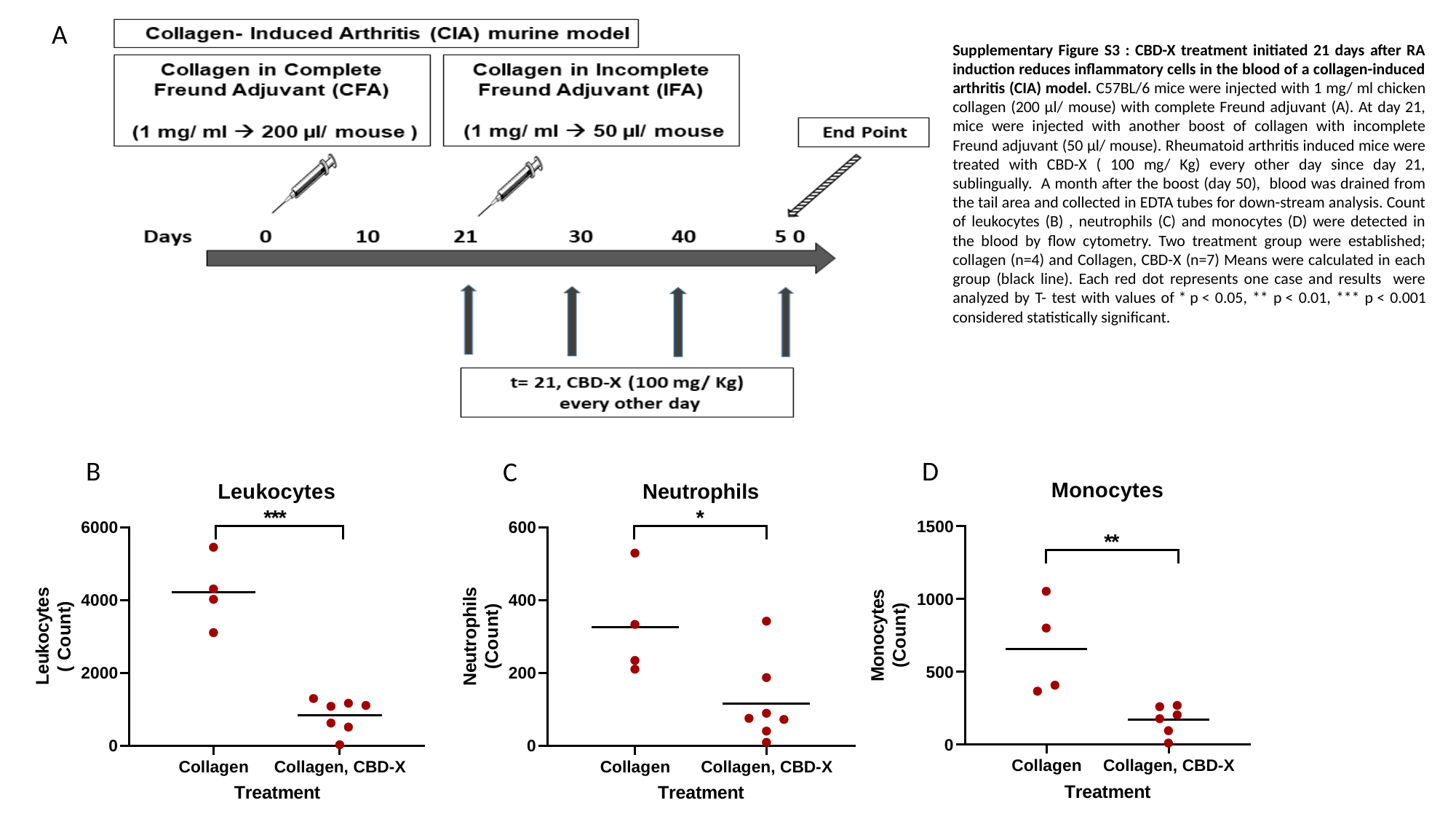

A
Supplementary Figure S3 : CBD-X treatment initiated 21 days after RA induction reduces inflammatory cells in the blood of a collagen-induced arthritis (CIA) model. C57BL/6 mice were injected with 1 mg/ ml chicken collagen (200 µl/ mouse) with complete Freund adjuvant (A). At day 21, mice were injected with another boost of collagen with incomplete Freund adjuvant (50 µl/ mouse). Rheumatoid arthritis induced mice were treated with CBD-X ( 100 mg/ Kg) every other day since day 21, sublingually. A month after the boost (day 50), blood was drained from the tail area and collected in EDTA tubes for down-stream analysis. Count of leukocytes (B) , neutrophils (C) and monocytes (D) were detected in the blood by flow cytometry. Two treatment group were established; collagen (n=4) and Collagen, CBD-X (n=7) Means were calculated in each group (black line). Each red dot represents one case and results were analyzed by T- test with values of * p < 0.05, ** p < 0.01, *** p < 0.001 considered statistically significant.
B
D
C

## Slide 4
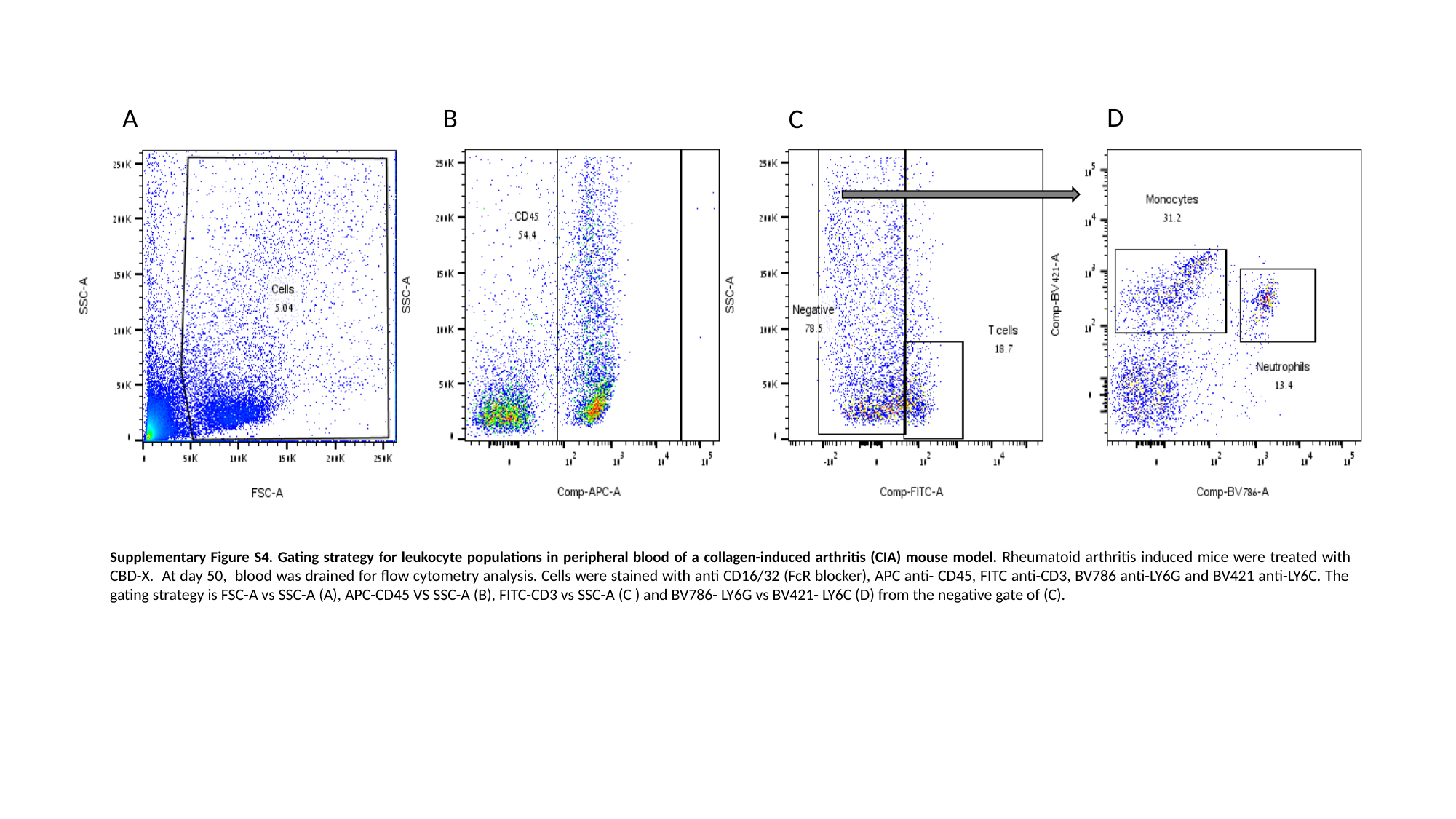

D
A
B
C
Supplementary Figure S4. Gating strategy for leukocyte populations in peripheral blood of a collagen-induced arthritis (CIA) mouse model. Rheumatoid arthritis induced mice were treated with CBD-X. At day 50, blood was drained for flow cytometry analysis. Cells were stained with anti CD16/32 (FcR blocker), APC anti- CD45, FITC anti-CD3, BV786 anti-LY6G and BV421 anti-LY6C. The gating strategy is FSC-A vs SSC-A (A), APC-CD45 VS SSC-A (B), FITC-CD3 vs SSC-A (C ) and BV786- LY6G vs BV421- LY6C (D) from the negative gate of (C).

## Slide 5
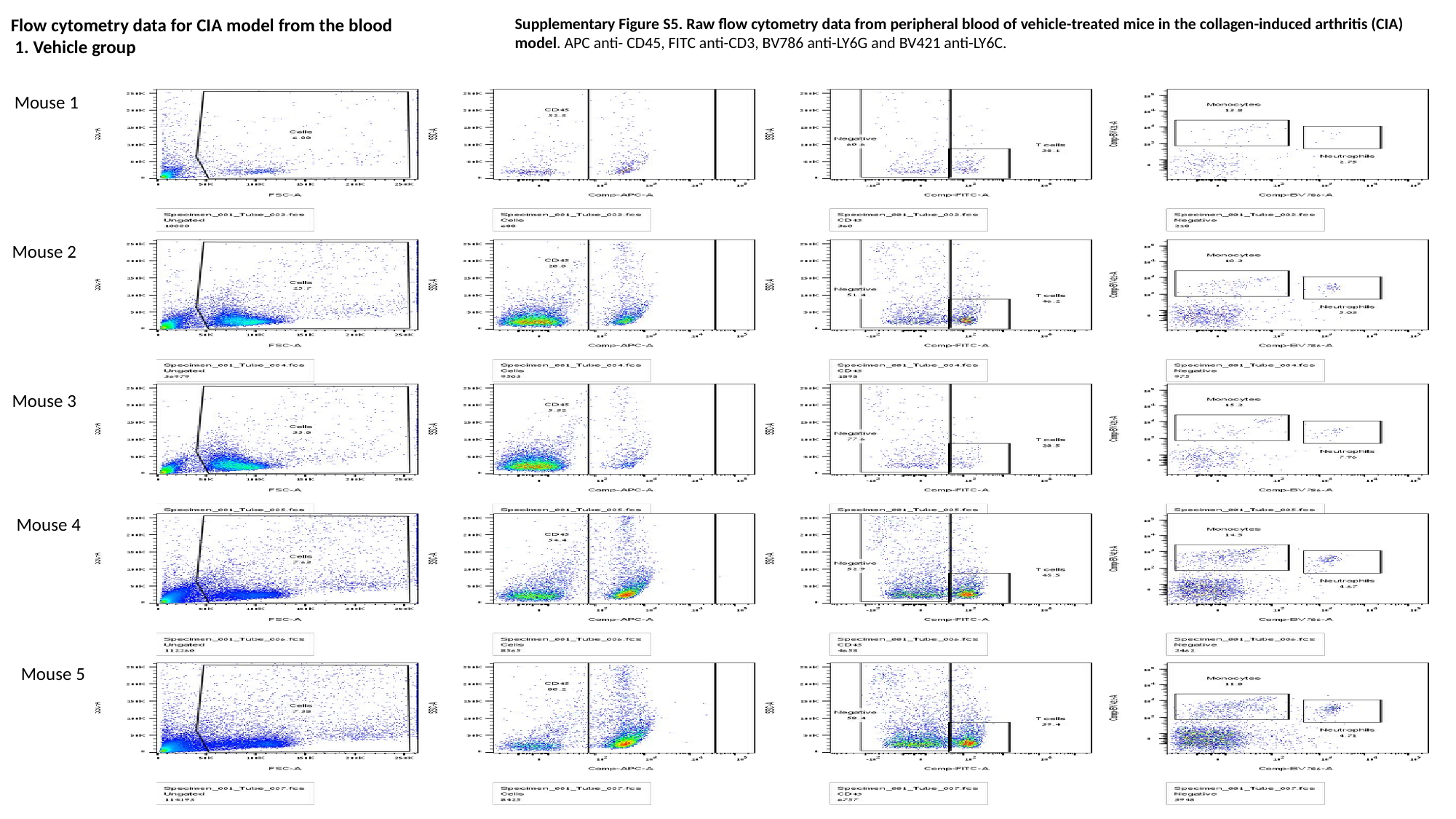

Flow cytometry data for CIA model from the blood
 1. Vehicle group
Supplementary Figure S5. Raw flow cytometry data from peripheral blood of vehicle-treated mice in the collagen-induced arthritis (CIA) model. APC anti- CD45, FITC anti-CD3, BV786 anti-LY6G and BV421 anti-LY6C.
Mouse 1
Mouse 2
Mouse 3
Mouse 4
Mouse 5

## Slide 6
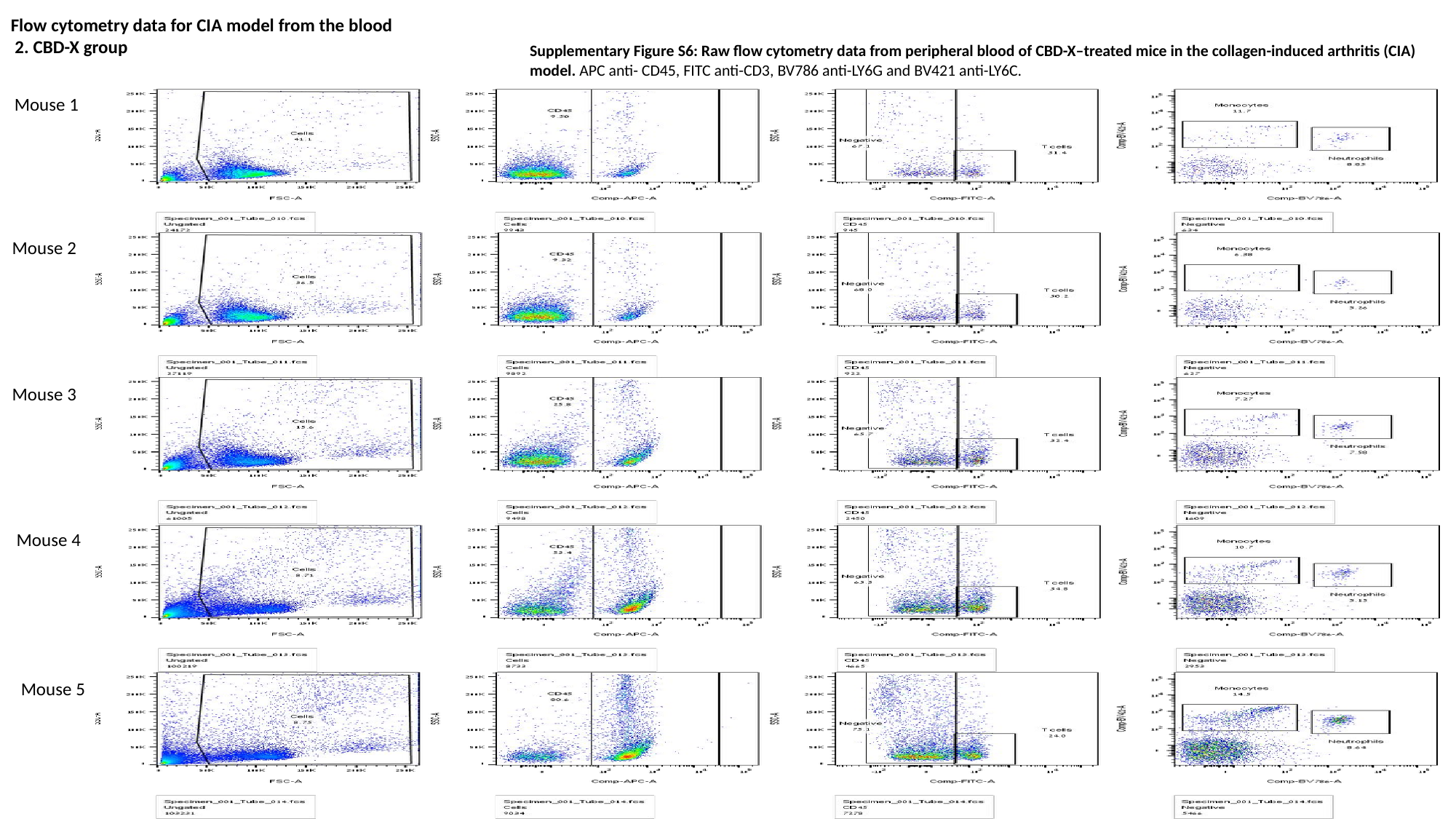

Flow cytometry data for CIA model from the blood
 2. CBD-X group
Supplementary Figure S6: Raw flow cytometry data from peripheral blood of CBD-X–treated mice in the collagen-induced arthritis (CIA) model. APC anti- CD45, FITC anti-CD3, BV786 anti-LY6G and BV421 anti-LY6C.
Mouse 1
Mouse 2
Mouse 3
Mouse 4
Mouse 5

## Slide 7
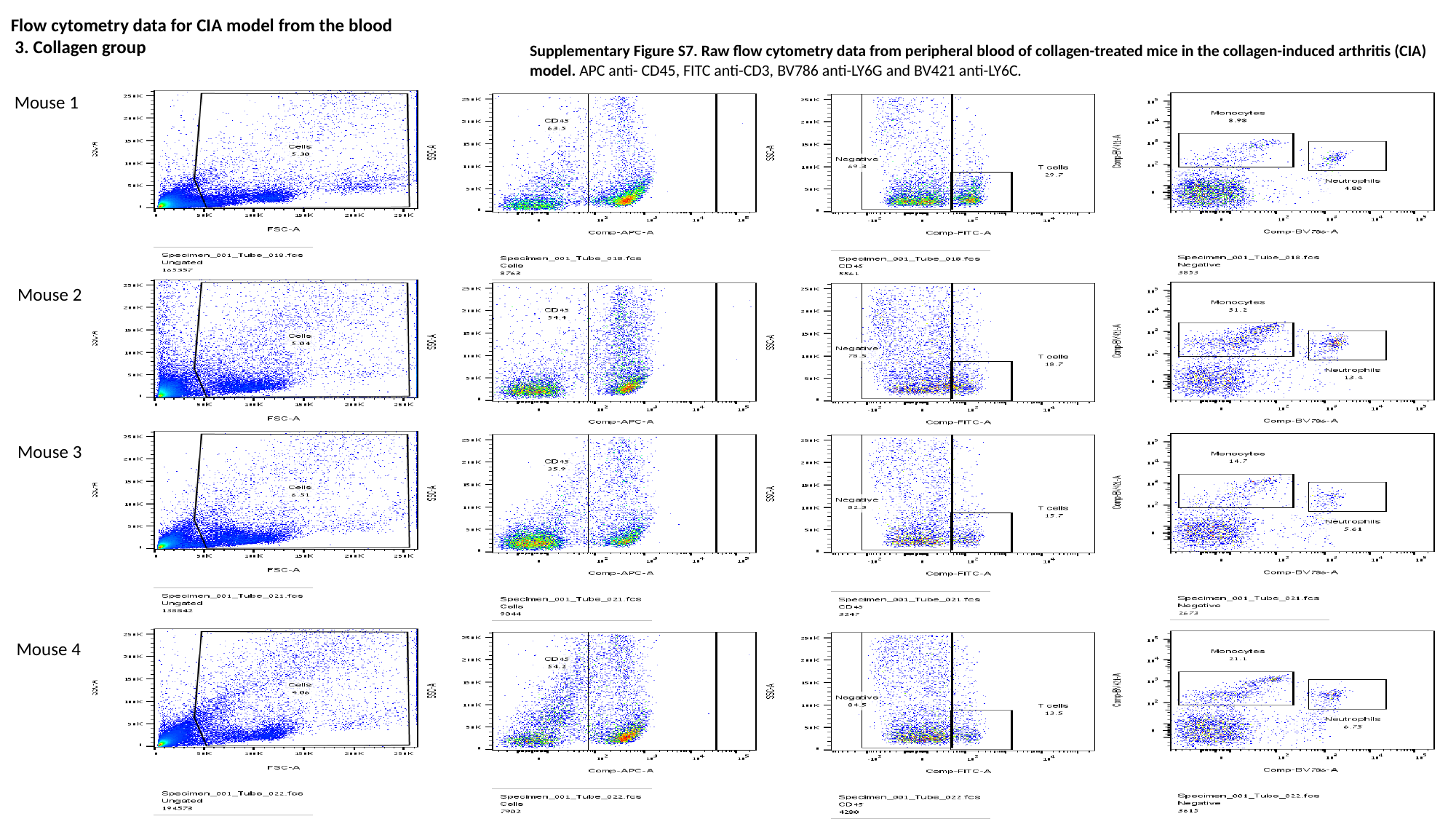

Flow cytometry data for CIA model from the blood
 3. Collagen group
Supplementary Figure S7. Raw flow cytometry data from peripheral blood of collagen-treated mice in the collagen-induced arthritis (CIA) model. APC anti- CD45, FITC anti-CD3, BV786 anti-LY6G and BV421 anti-LY6C.
Mouse 1
Mouse 2
Mouse 3
Mouse 4

## Slide 8
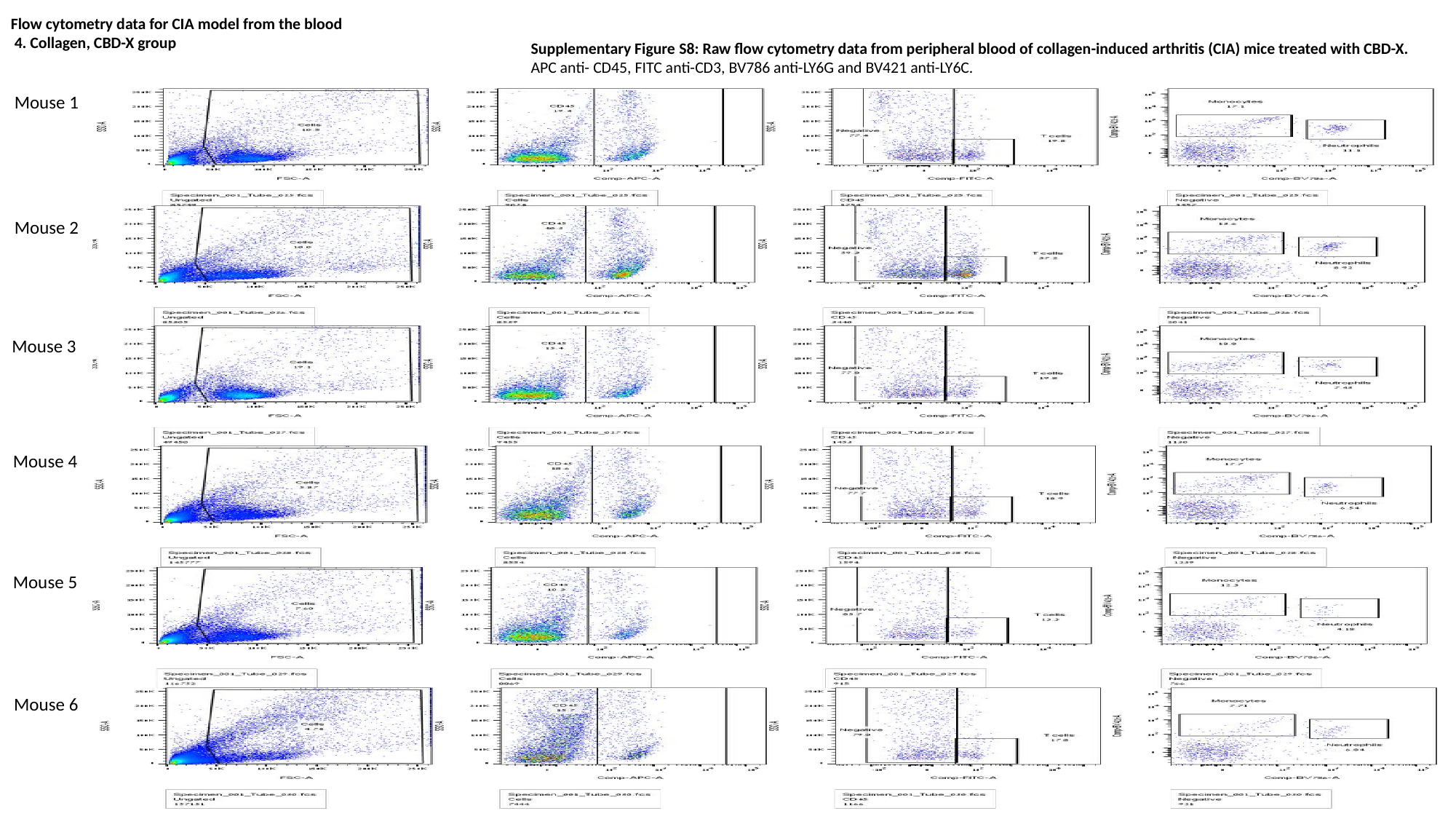

Flow cytometry data for CIA model from the blood
 4. Collagen, CBD-X group
Supplementary Figure S8: Raw flow cytometry data from peripheral blood of collagen-induced arthritis (CIA) mice treated with CBD-X. APC anti- CD45, FITC anti-CD3, BV786 anti-LY6G and BV421 anti-LY6C.
Mouse 1
Mouse 2
Mouse 3
Mouse 4
Mouse 5
Mouse 6

## Slide 9
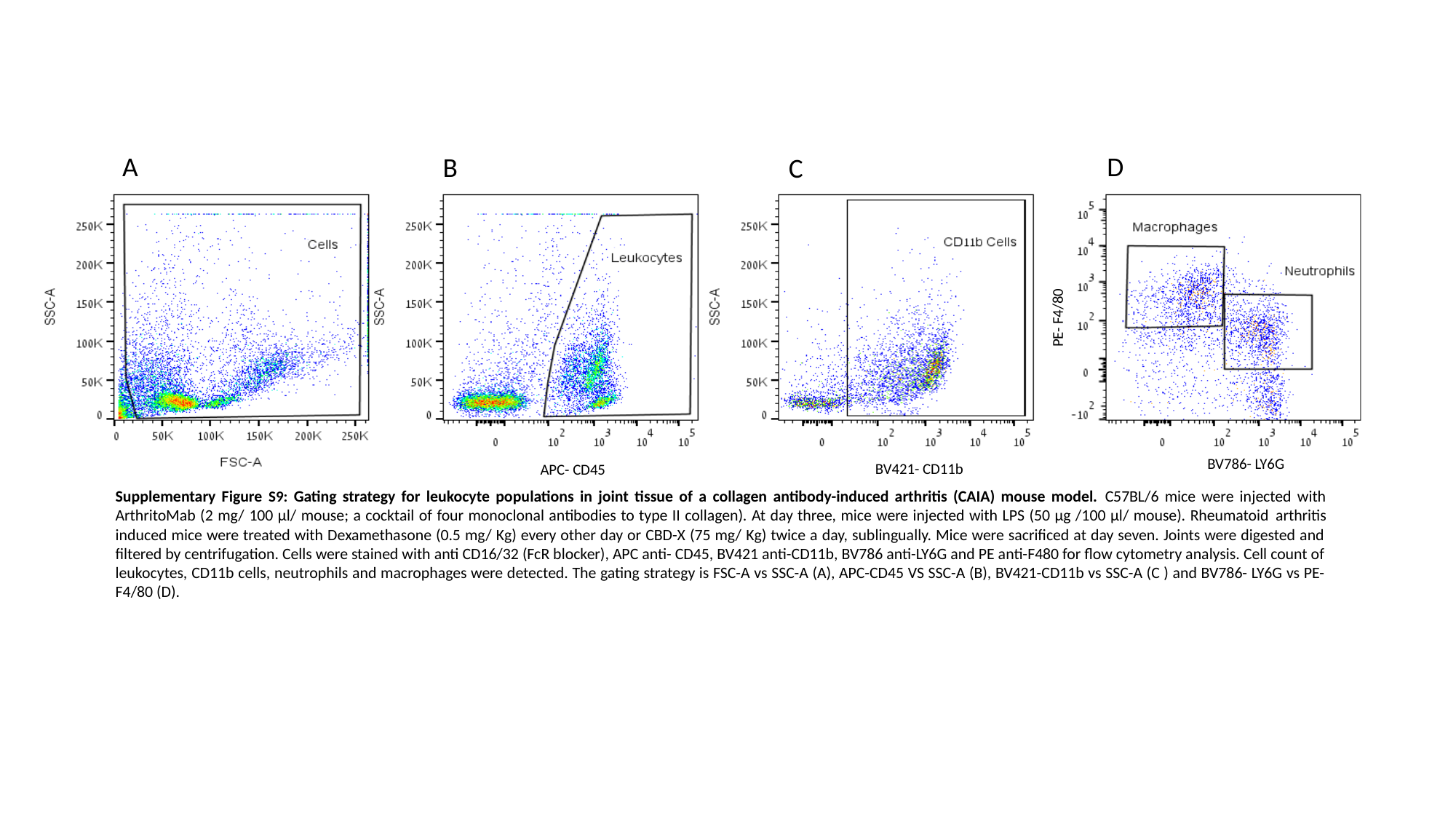

D
A
B
C
PE- F4/80
BV786- LY6G
BV421- CD11b
APC- CD45
Supplementary Figure S9: Gating strategy for leukocyte populations in joint tissue of a collagen antibody-induced arthritis (CAIA) mouse model. C57BL/6 mice were injected with ArthritoMab (2 mg/ 100 µl/ mouse; a cocktail of four monoclonal antibodies to type II collagen). At day three, mice were injected with LPS (50 μg /100 µl/ mouse). Rheumatoid arthritis induced mice were treated with Dexamethasone (0.5 mg/ Kg) every other day or CBD-X (75 mg/ Kg) twice a day, sublingually. Mice were sacrificed at day seven. Joints were digested and filtered by centrifugation. Cells were stained with anti CD16/32 (FcR blocker), APC anti- CD45, BV421 anti-CD11b, BV786 anti-LY6G and PE anti-F480 for flow cytometry analysis. Cell count of leukocytes, CD11b cells, neutrophils and macrophages were detected. The gating strategy is FSC-A vs SSC-A (A), APC-CD45 VS SSC-A (B), BV421-CD11b vs SSC-A (C ) and BV786- LY6G vs PE- F4/80 (D).

## Slide 10
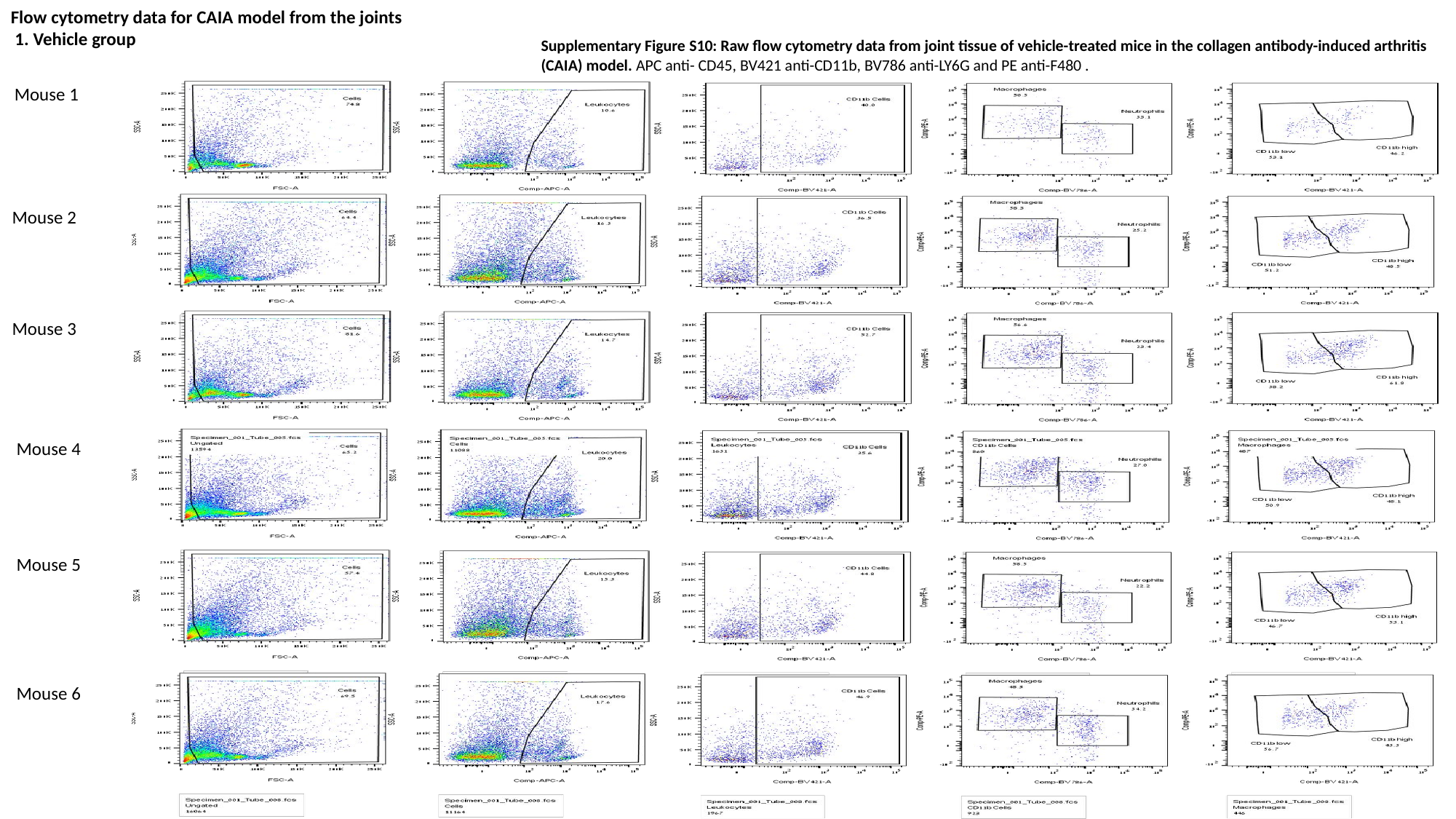

Flow cytometry data for CAIA model from the joints
 1. Vehicle group
Supplementary Figure S10: Raw flow cytometry data from joint tissue of vehicle-treated mice in the collagen antibody-induced arthritis (CAIA) model. APC anti- CD45, BV421 anti-CD11b, BV786 anti-LY6G and PE anti-F480 .
Mouse 1
Mouse 2
Mouse 3
Mouse 4
Mouse 5
Mouse 6

## Slide 11
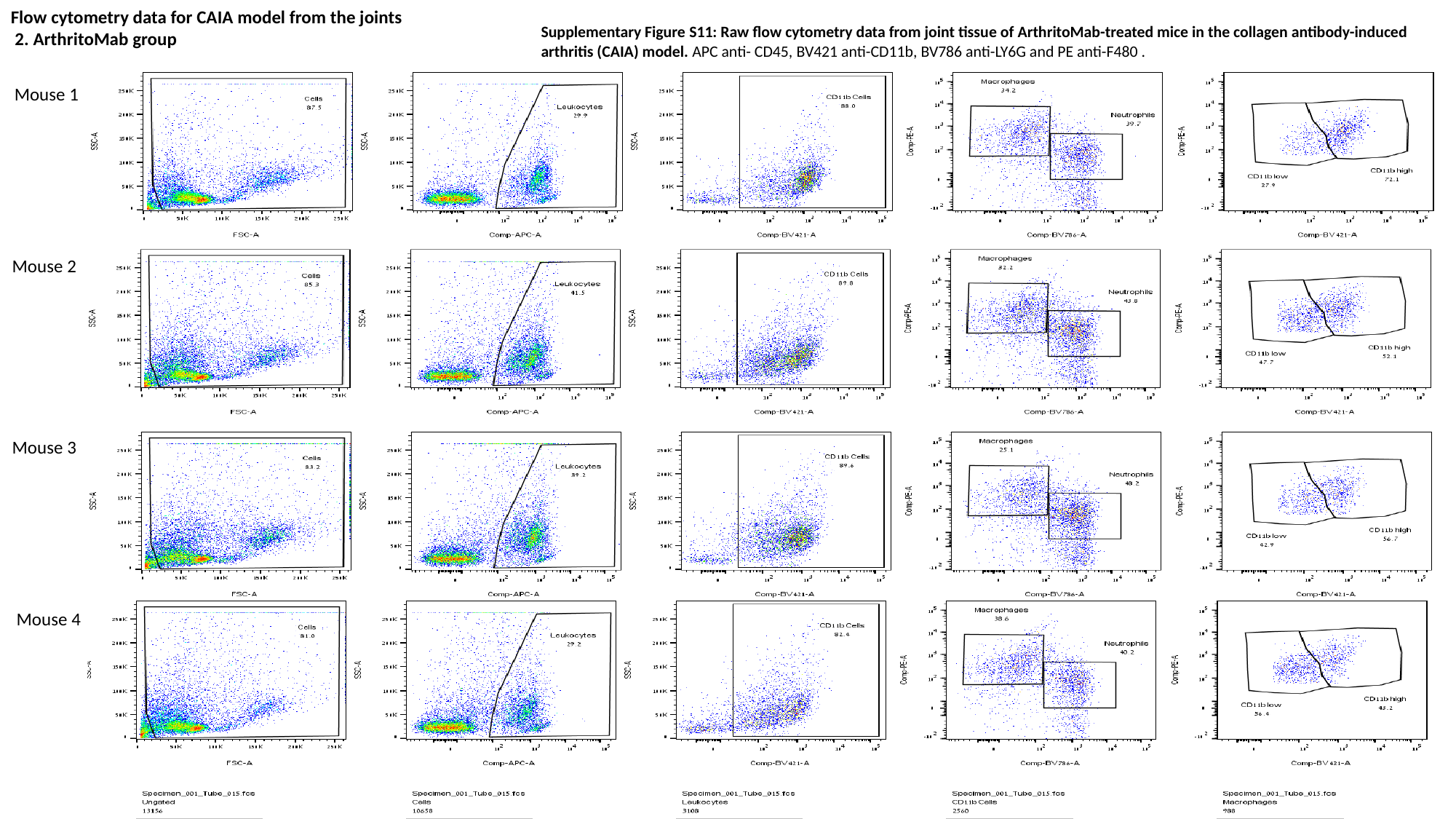

Flow cytometry data for CAIA model from the joints
 2. ArthritoMab group
Supplementary Figure S11: Raw flow cytometry data from joint tissue of ArthritoMab-treated mice in the collagen antibody-induced arthritis (CAIA) model. APC anti- CD45, BV421 anti-CD11b, BV786 anti-LY6G and PE anti-F480 .
Mouse 1
Mouse 2
Mouse 3
Mouse 4

## Slide 12
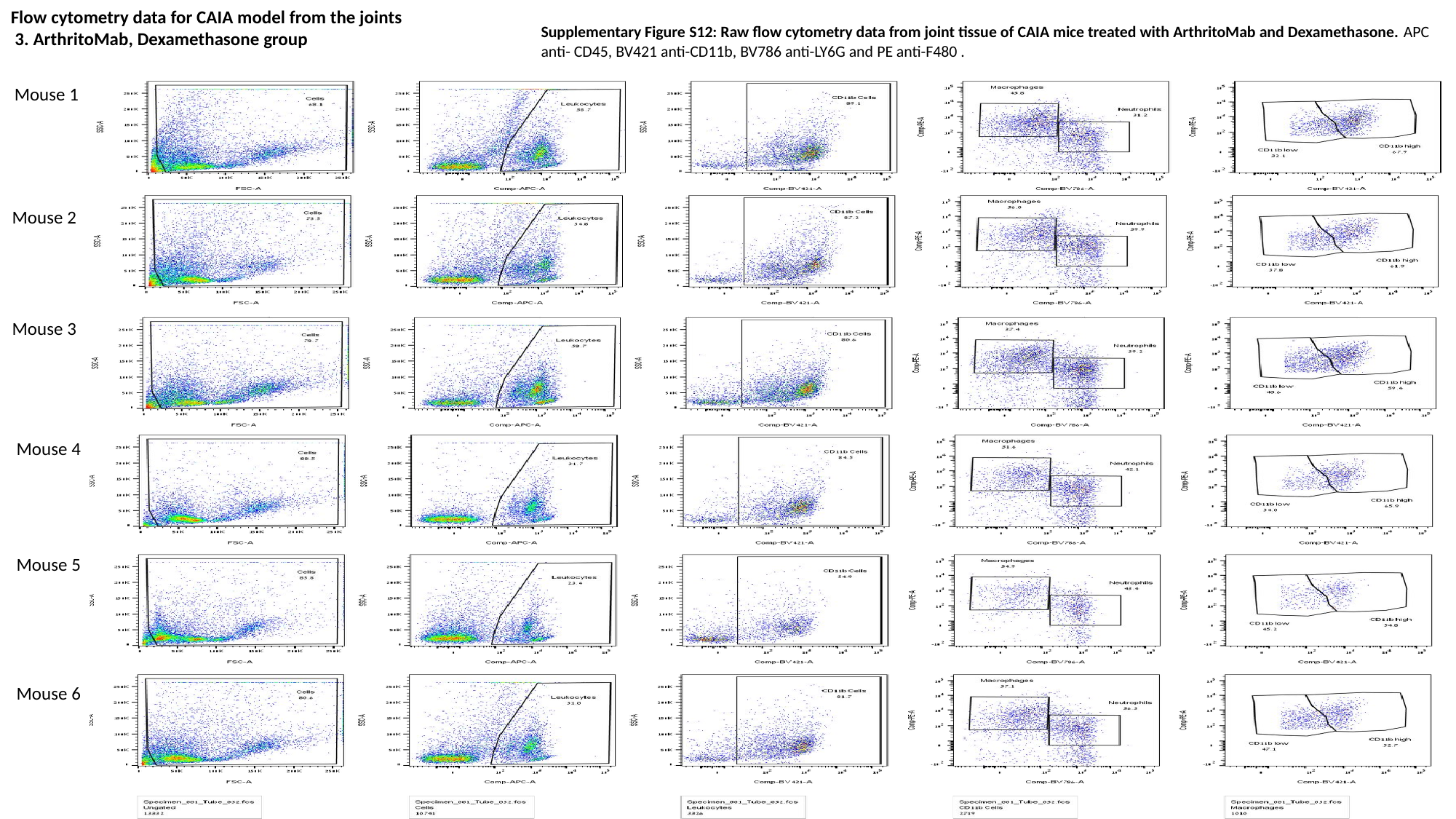

Flow cytometry data for CAIA model from the joints
 3. ArthritoMab, Dexamethasone group
Supplementary Figure S12: Raw flow cytometry data from joint tissue of CAIA mice treated with ArthritoMab and Dexamethasone. APC anti- CD45, BV421 anti-CD11b, BV786 anti-LY6G and PE anti-F480 .
Mouse 1
Mouse 2
Mouse 3
Mouse 4
Mouse 5
Mouse 6

## Slide 13
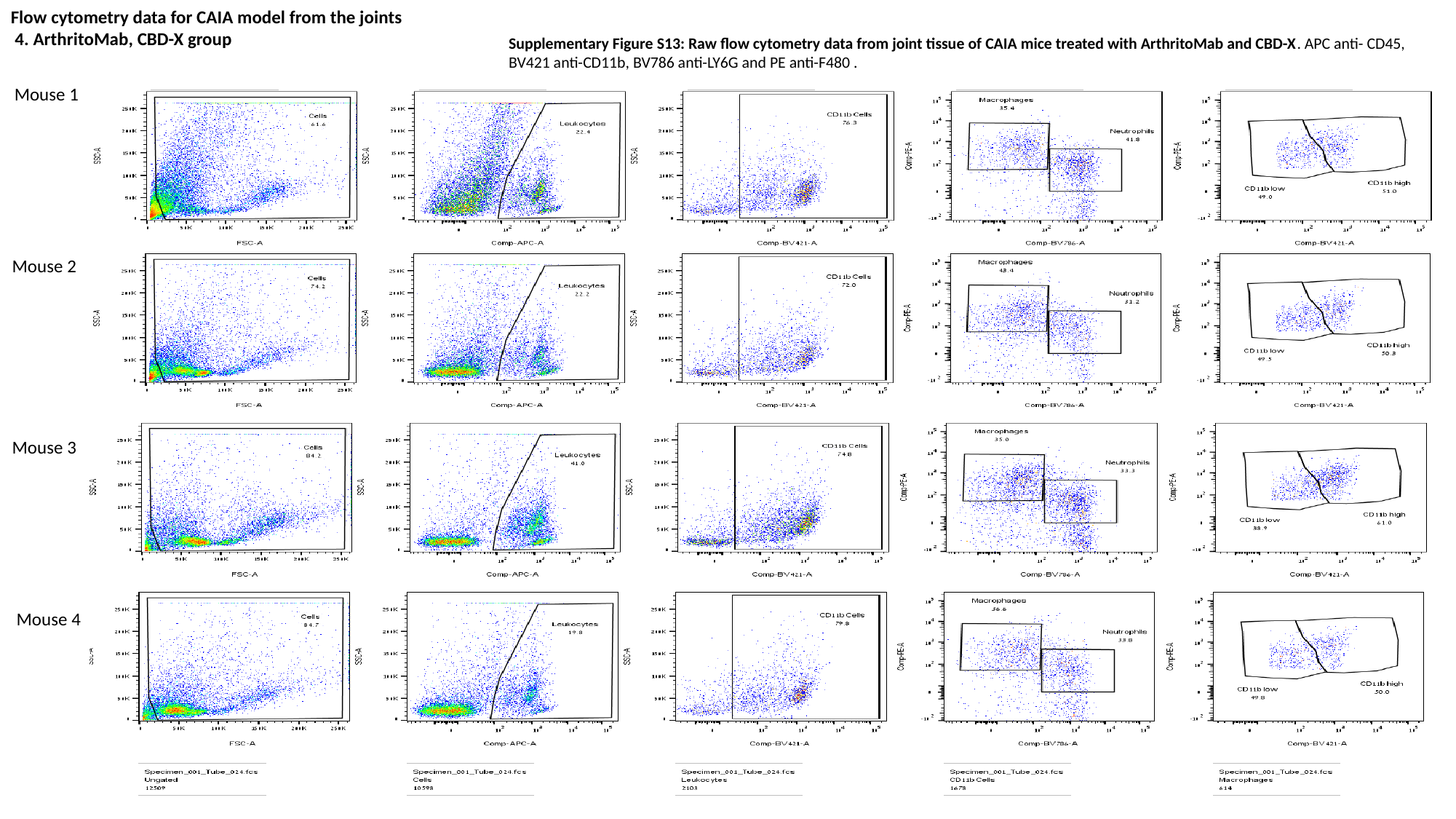

Flow cytometry data for CAIA model from the joints
 4. ArthritoMab, CBD-X group
Supplementary Figure S13: Raw flow cytometry data from joint tissue of CAIA mice treated with ArthritoMab and CBD-X. APC anti- CD45, BV421 anti-CD11b, BV786 anti-LY6G and PE anti-F480 .
Mouse 1
Mouse 2
Mouse 3
Mouse 4

## Slide 14
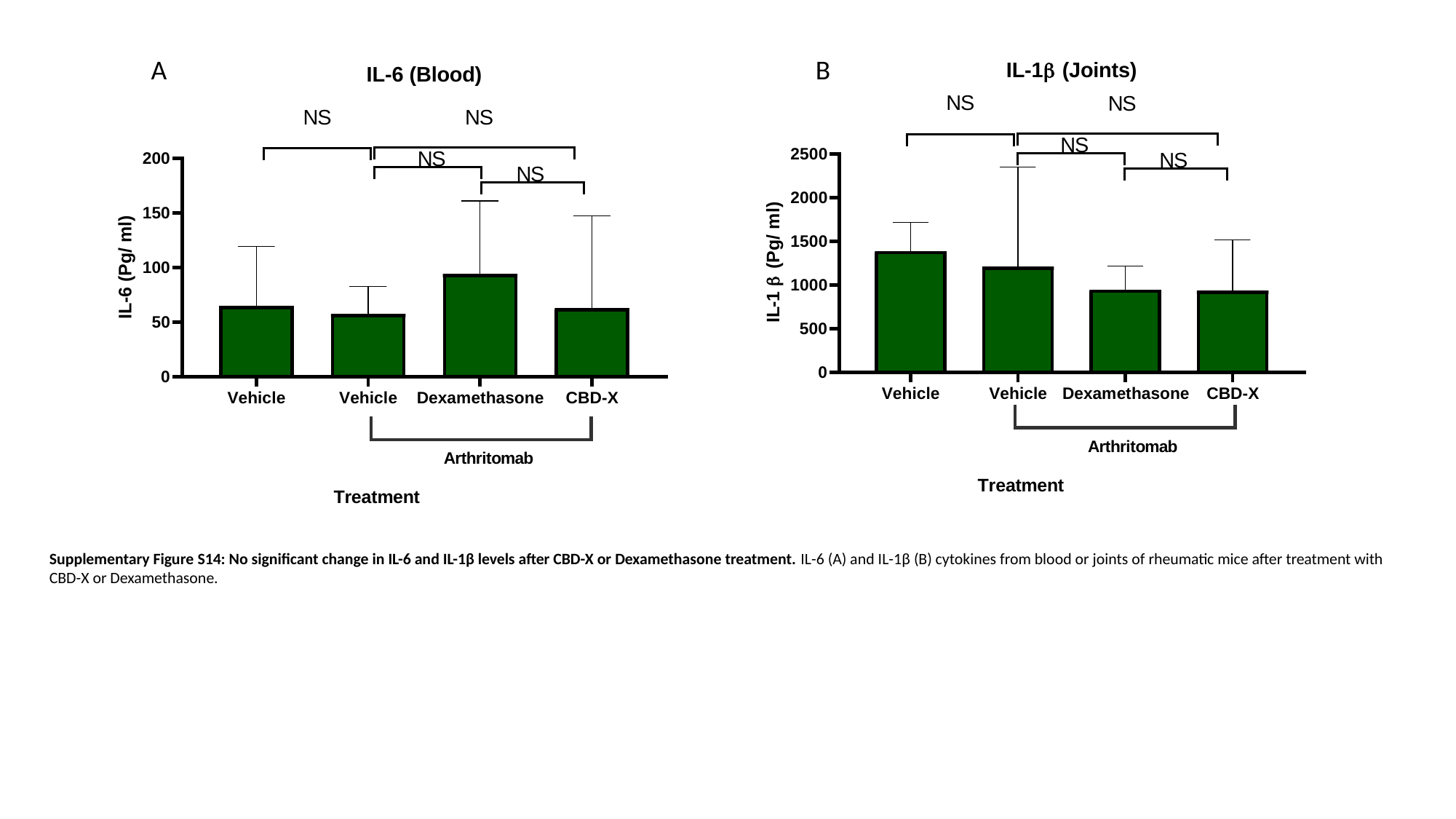

A
B
Supplementary Figure S14: No significant change in IL-6 and IL-1β levels after CBD-X or Dexamethasone treatment. IL-6 (A) and IL-1β (B) cytokines from blood or joints of rheumatic mice after treatment with CBD-X or Dexamethasone.

## Slide 15
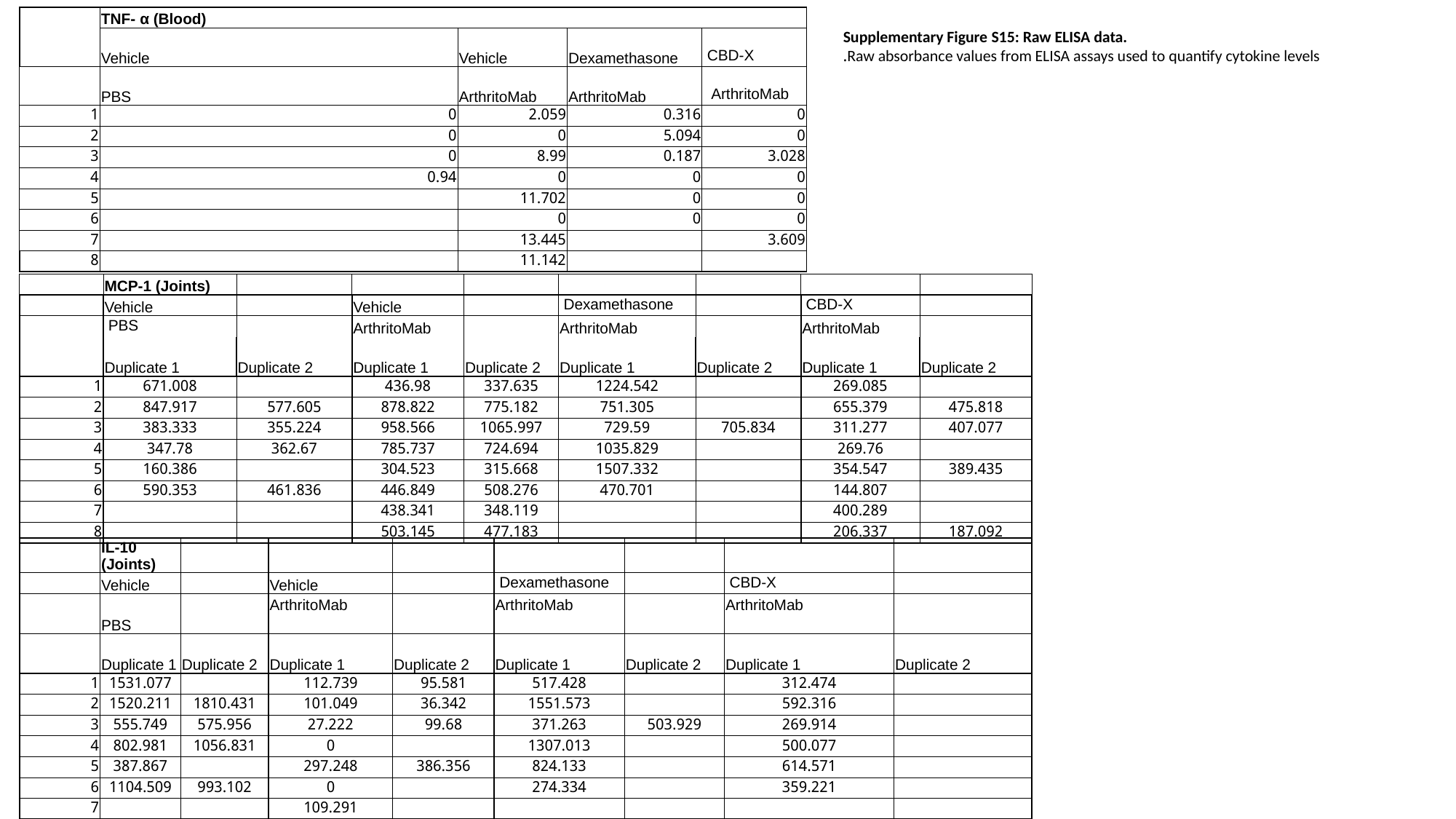

| | TNF- α (Blood) | | | |
| --- | --- | --- | --- | --- |
| | Vehicle | Vehicle | Dexamethasone | CBD-X |
| | PBS | ArthritoMab | ArthritoMab | ArthritoMab |
| 1 | 0 | 2.059 | 0.316 | 0 |
| 2 | 0 | 0 | 5.094 | 0 |
| 3 | 0 | 8.99 | 0.187 | 3.028 |
| 4 | 0.94 | 0 | 0 | 0 |
| 5 | | 11.702 | 0 | 0 |
| 6 | | 0 | 0 | 0 |
| 7 | | 13.445 | | 3.609 |
| 8 | | 11.142 | | |
Supplementary Figure S15: Raw ELISA data.
Raw absorbance values from ELISA assays used to quantify cytokine levels.
| | MCP-1 (Joints) | | | | | | | |
| --- | --- | --- | --- | --- | --- | --- | --- | --- |
| | Vehicle | | Vehicle | | Dexamethasone | | CBD-X | |
| | PBS | | ArthritoMab | | ArthritoMab | | ArthritoMab | |
| | Duplicate 1 | Duplicate 2 | Duplicate 1 | Duplicate 2 | Duplicate 1 | Duplicate 2 | Duplicate 1 | Duplicate 2 |
| 1 | 671.008 | | 436.98 | 337.635 | 1224.542 | | 269.085 | |
| 2 | 847.917 | 577.605 | 878.822 | 775.182 | 751.305 | | 655.379 | 475.818 |
| 3 | 383.333 | 355.224 | 958.566 | 1065.997 | 729.59 | 705.834 | 311.277 | 407.077 |
| 4 | 347.78 | 362.67 | 785.737 | 724.694 | 1035.829 | | 269.76 | |
| 5 | 160.386 | | 304.523 | 315.668 | 1507.332 | | 354.547 | 389.435 |
| 6 | 590.353 | 461.836 | 446.849 | 508.276 | 470.701 | | 144.807 | |
| 7 | | | 438.341 | 348.119 | | | 400.289 | |
| 8 | | | 503.145 | 477.183 | | | 206.337 | 187.092 |
| | IL-10 (Joints) | | | | | | | |
| --- | --- | --- | --- | --- | --- | --- | --- | --- |
| | Vehicle | | Vehicle | | Dexamethasone | | CBD-X | |
| | PBS | | ArthritoMab | | ArthritoMab | | ArthritoMab | |
| | Duplicate 1 | Duplicate 2 | Duplicate 1 | Duplicate 2 | Duplicate 1 | Duplicate 2 | Duplicate 1 | Duplicate 2 |
| 1 | 1531.077 | | 112.739 | 95.581 | 517.428 | | 312.474 | |
| 2 | 1520.211 | 1810.431 | 101.049 | 36.342 | 1551.573 | | 592.316 | |
| 3 | 555.749 | 575.956 | 27.222 | 99.68 | 371.263 | 503.929 | 269.914 | |
| 4 | 802.981 | 1056.831 | 0 | | 1307.013 | | 500.077 | |
| 5 | 387.867 | | 297.248 | 386.356 | 824.133 | | 614.571 | |
| 6 | 1104.509 | 993.102 | 0 | | 274.334 | | 359.221 | |
| 7 | | | 109.291 | | | | | |

## Slide 16
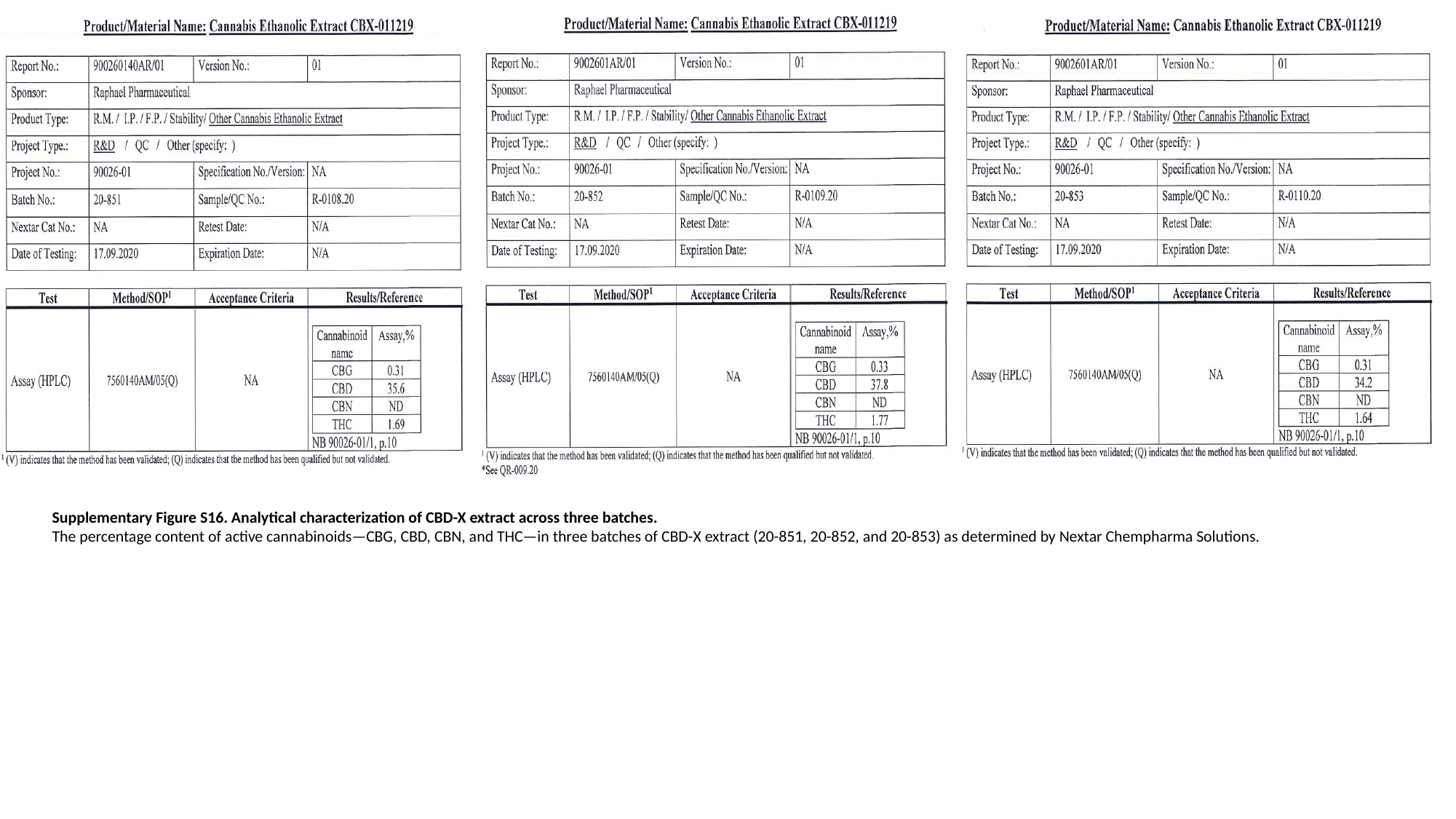

Supplementary Figure S16. Analytical characterization of CBD-X extract across three batches.
The percentage content of active cannabinoids—CBG, CBD, CBN, and THC—in three batches of CBD-X extract (20-851, 20-852, and 20-853) as determined by Nextar Chempharma Solutions.
